# Novel transketolase inhibitor oroxylin A suppresses the non‐oxidative pentose phosphate pathway and hepatocellular carcinoma tumour growth in mice and patient‐derived organoids

**DOI:** 10.1002/ctm2.1095

**Published:** 2022-10-31

**Authors:** Dan Jia, Chunliang Liu, Zhenyu Zhu, Yan Cao, Wen Wen, Zhanying Hong, Yue Liu, Erdong Liu, Long Chen, Chun Chen, Yanqiu Gu, Binghua Jiao, Yifeng Chai, Hong‐yang Wang, Jing Fu, Xiaofei Chen

**Affiliations:** ^1^ International Cooperation Laboratory on Signal Transduction Eastern Hepatobiliary Surgery Hospital Second Military Medical University/Naval Medical University Shanghai China; ^2^ School of Pharmacy Second Military Medical University/Naval Medical University Shanghai China; ^3^ National Center for Liver Cancer Second Military Medical University/Naval Medical University Shanghai China; ^4^ Department of Biochemistry and Molecular Biology College of Basic Medical Second Military Medical University/Naval Medical University Shanghai China; ^5^ Department of Pharmacy Shanghai Ninth People's Hospital School of Medicine of Shanghai Jiao Tong University Shanghai China

**Keywords:** metabolic reprogramming, non‐oxidative pentose phosphate pathway, oroxylin A, patient‐derived organoids, transketolase

## Abstract

**Background:**

Transketolase (TKT), a key rate‐limiting enzyme in the non‐oxidative branch of the pentose phosphate pathway (PPP), provides more than 85% of the ribose required for de novo nucleotide biosynthesis and promotes the development of hepatocellular carcinoma (HCC). Pharmacologic inhibition of TKT could impede HCC development and enhance treatment efficacy. However, no safe and effective TKT inhibitor has been approved.

**Methods:**

An online two‐dimensional TKT protein immobilised biochromatographic system was established for high‐throughput screening of TKT ligands. Oroxylin A was found to specifically bind TKT. Drug affinity responsive target stability, cellular thermal shift assay, surface plasmon resonance, molecular docking, competitive displacement assay, and site mutation were performed to identify the binding of oroxylin A with TKT. Antitumour effects of oroxylin A were evaluated in vitro, in human xenograft mice, diethylnitrosamine (DEN)‐induced HCC mice, and patient‐derived organoids (PDOs). Metabolomic analysis was applied to detect the enzyme activity. Transcriptome profiling was conducted to illustrate the anti‐HCC mechanism of oroxylin A. TKT knocking‐down HCC cell lines and PDOs were established to evaluate the role of TKT in oroxylin A‐induced HCC suppression.

**Results:**

By targeting TKT, oroxylin A stabilised the protein to proteases and temperature extremes, decreased its activity and expression, resulted in accumulation of non‐oxidative PPP substrates, and activated p53 signalling. In addition, oroxylin A suppressed cell proliferation, induced apoptosis and cell‐cycle arrest, and inhibited the growth of human xenograft tumours and DEN‐induced HCC in mice. Crucially, TKT depletion exerted identical effects to oroxylin A, and the promising inhibitor also exhibited excellent therapeutic efficacy against clinically relevant HCC PDOs.

**Conclusions:**

These results uncover a unique role for oroxylin A in TKT inhibition, which directly targets TKT and suppresses the non‐oxidative PPP. Our findings will facilitate the development of small‐molecule inhibitors of TKT and novel therapeutics for HCC.

## Graphical abstract

Oroxylin A directly targets transketolase (TKT) to suppress the non‐oxidative pentose phosphate pathway and activate p53 signalling, leading to growth inhibition of hepatocellular carcinoma (HCC) cells, tumours, and patient‐derived tumour organoids. This will facilitate the development of small‐molecule inhibitors of TKT and novel therapeutics for HCC.

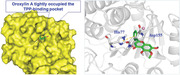

## 1 INTRODUCTION

Metabolic reprogramming is a hallmark of cancer cells, which prefers anaerobic glycolysis over mitochondrial oxidative phosphorylation for adenosine triphosphate (ATP) production.^1^, ^2^ Specifically, increased glucose oxidation is a feature of cancer cells, and these metabolic heterogeneous processes are regulated by various enzymes.^3^ Transketolase (TKT) is a key rate‐limiting enzyme in the non‐oxidative branch of the pentose phosphate pathway (PPP), which produces more than 85% of ribose‐5‐phosphate (R5P), an important precursor for DNA and RNA biosynthesis.^4^, ^5^ TKT mediates two reversible reactions in the non‐oxidative PPP: reversible conversion of R5P and xylulose‐5‐phosphate (Xu5P) to glyceraldehyde‐3‐phosphate (G3P) and sedoheptulose‐7‐phosphate (S7P), followed by conversion of Xu5P and erythrose‐4‐phosphate (E4P) to fructose‐6‐phosphate (F6P) and G3P.^6^

Three genes encoding TKT isozymes have been identified in the human genome: TKT and two TKT‐like genes (TKTL1 and TKTL2).^7^ TKT, rather than TKTL1 and TKTL2, was upregulated in cancers compared to non‐malignant tissues.^8^, ^9^ Upregulated TKT family genes have been identified in multiple types of cancer—including hepatocellular carcinoma (HCC), breast cancer, ovarian cancer, oesophageal cancer, lung cancer, and head‐and‐neck cancer, and is indicative of a poor prognosis. Thus, TKT is a potential therapeutic target for cancer therapy.^10^ To date, few inhibitors have been shown to inhibit TKT with low efficacy or unpredictable side effects in clinical trials.^11^ Therefore, highly potent and selective TKT inhibitors are urgently needed.

HCC, accounting for 80% of primary liver cancer, is the fifth most common cancer and the second leading cause of cancer‐related death worldwide.^12^ Patients with HCC have a 5‐year survival rate of <10%.^13^ Studies revealed that TKT promoted HCC development in both metabolic and non‐metabolic respects, and blockade of TKT sensitised cells to sorafenib, whereas TKT deficiency prevented DNA damage in the liver.^14^, ^15^, ^16^ Therefore, pharmacologic inhibition of TKT could impede HCC development and enhance treatment efficacy.

In this study, an online two‐dimensional TKT‐immobilised biological chromatography/C18 column/time‐of‐flight mass spectrometry system was established for high‐throughput screening of TKT ligands from herbal extracts, and oroxylin A was identified as a TKT ligand. By targeting TKT, oroxylin A inhibits the non‐oxidative PPP and HCC tumour growth in mice and patient‐derived organoids (PDOs). Our findings will facilitate the development of small‐molecule inhibitors of TKT and novel therapeutics for HCC.

## 2 MATERIALS AND METHODS

### 2.1 Chemicals and reagents

Oroxylin A was purchased from Shanghai Standard Technology Co., Ltd. (Shanghai, China; purity ≥ 98%), dissolved in dimethyl sulphoxide (DMSO) to 20 mM, and stored at −20°C. *Radix scutellariae* extraction was prepared according to our previously reported conditions with the concentrated decoction of 1 g·ml^−1^. Cell lines were purchased from the Cell Bank of Shanghai Branch of the Chinese Academy of Sciences and authenticated by Shanghai Biowing Biotechnology Co. Ltd. (Shanghai, China) using short tandem repeat markers. Dulbecco's modified Eagle's medium (DMEM) and phosphate‐buffered saline (PBS) were purchased from Hyclone (Thermo Fisher, Waltham, MA). Fetal bovine serum (FBS) was obtained from Gibco (Grand Island, NY). DMSO, penicillin, streptomycin, and trypsin were purchased from Gibco‐BRL Co. (Gaithersburg, MD). Ultrapure water was prepared using a Milli‐Q Academic A10 water purification system (Millipore, Bedford, MA). All chemicals were purchased from Sigma‐Aldrich (St. Louis, MO) unless otherwise noted and were of analytical grade. Antibodies against TKT, glyceraldehyde‐3‐phosphate dehydrogenase (GAPDH), and β‐actin were obtained from Santa Cruz Biotechnology (Santa Cruz, CA). Antibodies against p53, cleaved‐caspase‐3, cleaved‐caspase‐9, Bcl‐2, Bax, and p‐p53 were acquired from Cell Signalling Technologies (Danvers, MA). IRDye800‐conjugated anti‐mouse and anti‐rabbit secondary antibodies were obtained from Rockland Immunochemicals, Inc. (Philadelphia, PA).

### 2.2 Cell culture

Cells were cultured in DMEM medium supplemented with 10% FBS, 100 U·ml^−1^ benzylpenicillin, and 100 μg·ml^−1^ streptomycin. Cells were grown at 37°C in a humidified atmosphere with 5% CO_2_, and were harvested from exponentially growing cultures. Cell proliferation, apoptosis and cycle, NADPH/:nicotinamide adenine dinucleotide phosphate (NADP^+^), and glucose assays are detailed in the Supporting Information.

### 2.3 Two‐dimensional TKT protein biological chromatography

Briefly, a TKT biological chromatographic column packed with mercaptopropyltrimethoxysilane‐modified TKT‐immobilised silica stationary phase was prepared. Binding ligands to TKT from *Scutellariae Radix* extracts were screened by the online comprehensive two‐dimensional TKT biological chromatography/high performance liquid chromatography/time‐of‐flight mass spectrometry system equipped with an Agilent 1200 series HPLC system, a 6220 TOF mass spectrometer, and an Agilent MassHunter Workstation (Agilent Technologies). Detailed experimental information including column packing, screening, validation, data processing, and signal deduction were performed according to our previous studies.^17^, ^18^

### 2.4 Drug affinity responsive target stability assay

The procedure was performed as reported previously.^19^ Cells were lysed and treated with various concentrations of oroxylin A, followed by digestion with pronase and addition of sodium dodecyl sulphate‐polyacrylamide gel electrophoresis (SDS‐PAGE) loading buffer. Next, the samples were subjected to SDS‐PAGE and silver staining. The oroxylin A‐protected candidate proteins were identified by matrix‐assisted laser desorption/ionisation time‐of‐flight mass spectrometry (MALDI‐TOF‐MS) and confirmed by western blotting.

### 2.5 Western blotting

Whole‐cell protein samples (25 μg) were separated by SDS‐PAGE, transferred to polyvinylidene difluoride membranes (0.45 μm), and blocked with 5% skimmed milk in Tris‐buffered saline containing 0.05% Tween‐20 at room temperature for 1 h. The membranes were incubated with primary antibodies against TKT (1:2000), p53 (1:2000), cleaved‐caspase‐3 (1:800), cleaved‐caspase‐9 (1:800), Bcl‐2 (1:1000), Bax (1:1000), p‐p53 (1:800), β‐actin (1:2000), and GAPDH (1:3000) at 4°C overnight, and then with IRDye^®^ 800CW goat anti‐rabbit or IRDye^®^ 680RD goat anti‐mouse secondary antibody (1:10000) in dark at room temperature for 2 h. GAPDH or β‐actin was used as the loading control. Band intensity was measured using an Odyssey Fc detection system (LI‐COR, Lincoln, NE) and analysed by scanning densitometry using a Tanon Image System (Tanon, Shanghai, China).

### 2.6 Cellular thermal shift assay

Cellular thermal shift assay (CETSA) was performed as reported previously, with minor modifications.^20^ Briefly, HepG2 cells were treated with 0 or 50 μM oroxylin A for 2 h, washed with PBS and lysed. Total proteins suspended in PBS supplemented with protease inhibitor cocktail were divided into 10 equal parts, which were heated at 48, 51, 54, 57, 60, 63, 66, 69, 72, and 75°C for 3 min and frozen in liquid N_2_ for 3 min. Each sample underwent three freeze–thaw cycles. The corresponding loading buffer was added and the proteins were analysed by western blotting.

### 2.7 Surface plasmon resonance analysis

Purified human TKT protein (50 g·ml^−1^) was injected onto a HisCap Sensor Chip for immobilisation. Various concentrations of oroxylin A in running buffer (1× PBS, 0.5% DMSO) were passed over the chip to produce response signals. The association and dissociation rate constants were calculated using GE Biacore T2000 Evaluation software. The ratio of the association and dissociation rate constants was determined as the binding affinity.

### 2.8 Molecular docking

Molecular docking was performed using LeDock software (http://www.lephar.com) and the crystal structure of human TKT was downloaded from the Protein Data Bank (PDB code 3MOS).^21^

### 2.9 Xenograft studies

All animal experiments were performed according to the relevant national and local guidelines. Male BALB/c nude mice weighting 18‐22 g was purchased from Shanghai Laboratory Animal Co. (Shanghai, China) and raised in a pathogen‐free environment (23 ± 2°C and 55 ± 5% humidity). After adaptive feeding for 24 h, the mice were inoculated subcutaneously with 5 × 10^6^ cells. After 7 days, tumour sizes were determined using micrometre callipers and the following formula: volume = (width^2^ × length ÷ 2). Mice with tumour volumes of 150‐250 mm^3^ were randomly divided into five groups (7 mice/group): saline control group, positive control group (cyclophosphamide 20 mg·kg^−1^ once every 2 days), and oroxylin A 20, 40, and 80 mg·kg^−1^·day^−1^ groups. The treatments were administered intraperitoneally for 12 days and tumour size was measured once every 3 days. Finally, the mice were euthanised and tumours were segregated and weighed.

### 2.10 Diethylnitrosamine model of hepatocellular carcinoma

C57BL/6J mice were administered diethylnitrosamine (DEN) (25 mg·kg^‐1^; Sigma‐Aldrich) at 15 days after birth, and carbon tetrachloride (CCL_4_) solution after 2 weeks (olive oil = 1:9, 4 ml· kg^−1^) once a week for 5 months. DEN and CCL_4_ were delivered intraperitoneally. Next, mice were randomly divided into two groups (5 mice/group): a saline control group and 80 mg·kg^−1^ oroxylin A‐treated group. Treatments were administered intraperitoneally once every 2 days for 5 weeks. Finally, the mice were euthanised and their liver tissues were harvested.

### 2.11 Transketolase activity determination

TKT catalytic activity was determined based on the rate of NAD^+^ reduction in the presence of G3P dehydrogenase, with Xu5P and R5P as substrates.^22^ The reaction was initiated by the addition of 5 μg TKT and oroxylin A. The decrease in NADH was followed for 1 h at room temperature at 340 nm on a microplate reader (Bio‐Rad, Hercules, CA).

### 2.12 Targeted quantitative metabolites analysis

When cells in 100 mm plates reached approximately 70% confluence, the supernatant was replaced by fresh medium containing oroxylin A. After 24 h of incubation, cells were harvested for ultra‐performance liquid chromatography/quadrupole time‐of‐flight mass spectrometry (UPLC‐QTOF‐MS). The sample preparation and analysis procedures are detailed in Supporting Information.

### 2.13 Establishment of stable TKT‐knockdown cell lines

To establish stable TKT‐knockdown cell lines, lentivirus containing short hairpin RNA (shRNA) sequences against the TKT sequence (sh‐TKT‐1: GCCATCATCTATAACAACAAT; sh‐TKT‐2: GCATCTATAAGCTGGACAA; sh‐TKT‐3: GCCGCCAATACAAAGGGTA) and NC (sh‐NC: TTCTCCGAACGTGTCACGT) were purchased from Obio (Shanghai, China). The lentivirus was transfected into HepG2 and SMMC‐7721 cells. Empty lentiviral vector was used as negative control. Stable clones were selected with 6 μg·ml^−1^ puromycin for 2 weeks. TKT expression in stably transfected clones was validated by western blotting.

### 2.14 Culture of human liver tumour organoids

Fresh tissue samples were obtained from Eastern Hepatobiliary Surgery Hospital (Shanghai, China) with informed consent from patients. The study was approved by the Ethics Committee of Eastern Hepatobiliary Surgery Hospital. The tissue samples were immediately transported to the laboratory on ice. A small portion of each tumour specimen was fixed in formalin and paraffin embedded for histochemical analysis. The remainder of the tissue was dissociated and processed for organoid culture. Briefly, the tumour tissues were washed twice with PBS, minced into 1 mm^3^ pieces with scissors, and incubated at 37°C in digestion solution on an orbital shaker for 1‐2 h, until no visible cell mass remained. The digestion solution was composed of DMEM containing 4 mg·ml^−1^ collagenase D, 10 μM Y27632, and 1× primocin. Digestion was stopped by adding cold termination medium (DMEM with 1% penicillin/streptomycin, 1× primocin, 10 μM Y27632, and 10% FBS). The cell suspension was centrifuged for 5 min at 300‐400 g after filtering through a 70‐μm nylon cell strainer and washed twice with cold Advanced DMEM/F12. The cells were resuspended in cold human liver organoid medium (Advanced DMEM/F12 supplemented with 1% penicillin/streptomycin, 1% GlutaMAX‐I, 10 mM HEPES, 1× primocin, 1:50 B27 supplement, 1.25 mM *N*‐acetyl‐l‐cysteine, 50 ng·ml^−1^ recombinant mouse EGF, 10 μM Y27632, 500 nM A8301, 100 ng·ml^−1^ recombinant human FGF10, 1 ng·ml^−1^ recombinant human FGF‐basic, 10 μM forskolin, 25 ng·ml^−1^ recombinant human HGF, 10 mM nicotinamide, 5% (v/v) Noggin conditioned medium, 3 nM dexamethasone, and 10 nM recombinant human [Leu15]‐gastrin‐I), mixed with Matrigel, and seeded in a 24‐multiwell plate at 37°C for 1 h. After matrix polymerisation, human liver organoid medium was added to each well. The culture was changed every 3‐4 days. After 1‐2 weeks, the organoids were repeatedly blown with a pipette to disperse the cells, and subsequently replanted into Matrigel at a ratio of 1:2.

Tumour organoids were digested into single cells in TrypLE digestion solution (37°C, 5 min). Seven microliters of culture (1 × 10^5^ cells/ml) was seeded in a 96‐well plate and incubated at 37°C for 1 h. The medium was changed every 3‐4 days and organoid viability was assayed using Cell Titer‐Glo (Promega, Madison, WI) after 14 days.

### 2.15 Histology and immunohistochemistry

Tumour tissue and organoids were fixed with 4% paraformaldehyde (PFA), embedded in paraffin, sectioned (3‐5 μm thickness), and stained with haematoxylin and eosin (H&E) or processed for immunohistochemistry. Sections were deparaffinised, rehydrated, and stained following standard immunohistochemistry protocols using a primary anti‐TKT monoclonal antibody (1:200).

### 2.16 p53 signalling RT‐PCR array analysis

The RT‐PCR array experiments were conducted at Wcgene Biotechnology Corporation (Shanghai, China) using total RNA from TKT‐knockdown and control HepG2 cells. Data were analysed using Wcgene Biotech software. Genes with expression fold changes of more or less than 2.0 were considered to be of biological significance.

### 2.17 Statistical analysis

Statistical analysis was performed using GraphPad Prism 8.0 software. The band intensities of western blotting were analysed by Image J software. Data were expressed as mean ± SD and *t*‐test were performed using GraphPad. *p* values < 0.05 were regarded as statistically significant.

## 3 RESULTS

### 3.1 Screening and identification of oroxylin A as a transketolase ligand

**FIGURE 1:**
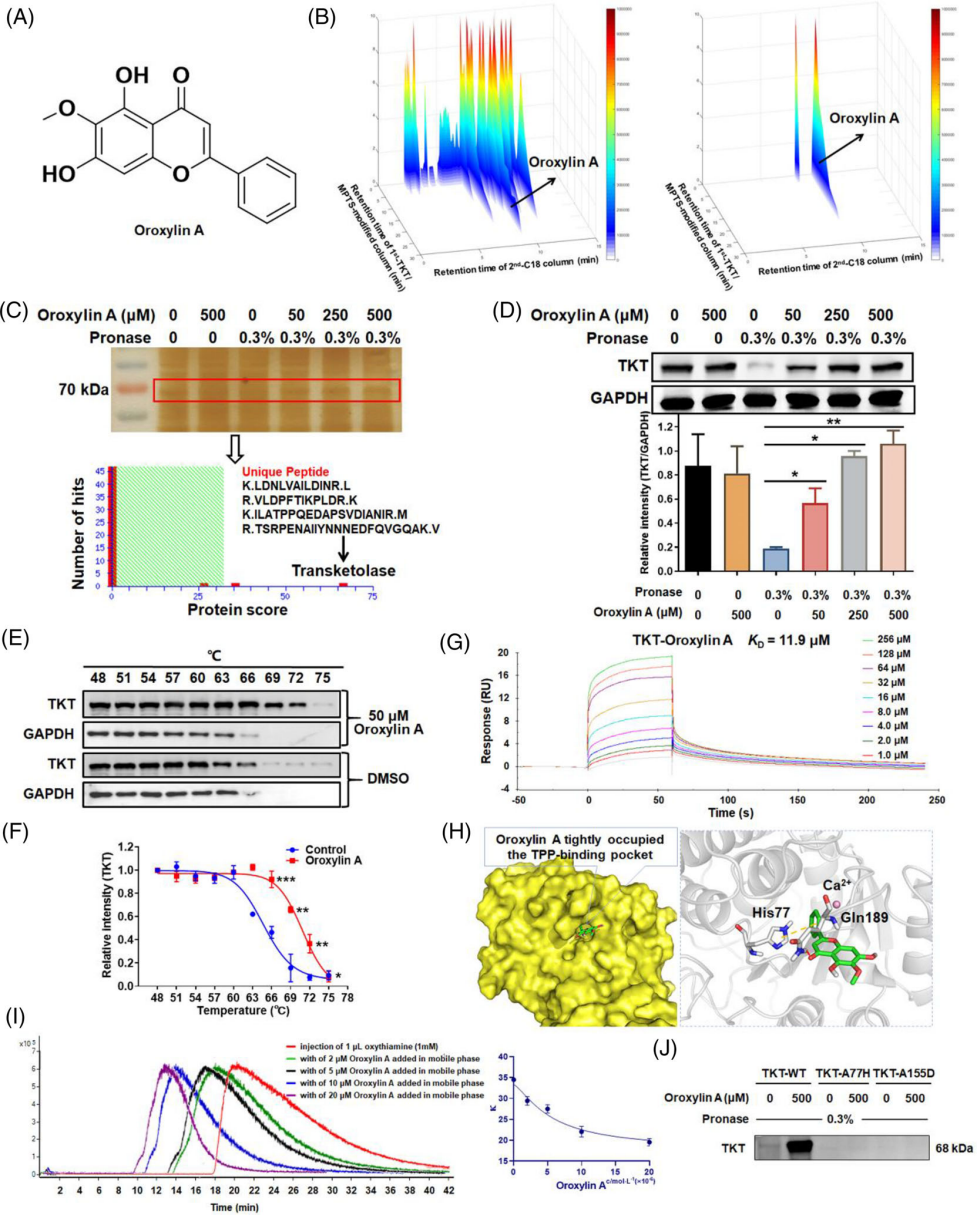
Screening and identification of oroxylin A as a transketolase (TKT) ligand. (A) Molecular structure of oroxylin A. (B) Rapid screening of oroxylin A as a novel TKT ligand using the 2D TKT protein biological chromatography/C18 column/time‐of‐flight mass spectrometry system. Typical 3D plots of *Radix scutellariae* extraction (left panel) and authentic standard of oroxylin A (right panel). (C) Oroxylin A protects TKT from proteolysis by drug affinity responsive target stability assay (DARTS) analysis. Samples were resolved by sodium dodecyl sulphate‐polyacrylamide gel electrophoresis, visualized by silver staining (upper panel), and identified by mass spectrometry (lower panel). (D) Samples were probed with an anti‐TKT antibody using GAPDH as the control. (E) Western blotting of the supernatant of HepG2 cell lysate incubated with 50 μM oroxylin A or dimethyl sulphoxide after heating and centrifugation using cellular thermal shift assay (CETSA). (F) Quantification of western blotting bands (mean ± SD, *n* = 3). (G) Surface plasmon resonance (SPR) analysis of oroxylin A and TKT immobilised on a chip (equilibrium dissociation constant = 11.9 ± 8.4 μM; *n* = 3). (H) Predicted binding mode of oroxylin A in the active site of human TKT (PDB code 3MOS). The protein is shown as a surface with carbon and hydrogen in grey, oxygen in red, and nitrogen in blue. The ligand is shown as sticks with carbons in cyan. Blue dashed lines are hydrogen bonds. (I) Left panel: Chromatograms of oxythiamine on TKT protein columns with various concentrations (0, 2, 5, 10, and 20 μM) of oroxylin A added to the mobile phase. Right panel: Competitive displacement assay of oxythiamine on TKT protein columns (presented as *Κ* values) with various concentrations (0, 2, 5, 10, and 20 μM) of oroxylin A added to the mobile phase. (J) Protection effects of oroxylin A on wild‐type TKT protein (TKT‐WT), histidine 77 site mutated TKT protein (TKT‐A77H), and aspartate 155 site mutated TKT protein (TKT‐A155D) using DARTS.

There are no effective TKT inhibitors for cancer therapy. Here, using an online 2D TKT‐immobilised biological chromatography/C18 column/time‐of‐flight mass spectrometry system, oroxylin A (Figure 1A), was screened from *R. scutellariae* (Figure 1B, left panel) and validated using standards (Figure 1B, right panel). Next, drug affinity responsive target stability assay (DARTS) was applied to identify the protein(s) directly binding oroxylin A. Silver staining showed a strong band at around 70 kDa in the lysate of oroxylin A‐treated cells, the intensity of which increased in a concentration‐dependent manner (Figure 1C, upper panel). MALDI‐TOF‐MS identified four unique peptides of TKT with the highest confidence (Figure 1C, lower panel), indicating that oroxylin A protected and enriched TKT during proteolysis. The results were confirmed by immunoblotting using an anti‐TKT monoclonal antibody (Figure 1D).

CETSA assays showed the thermal stability of TKT in oroxylin A‐treated cells was increased compared to control cells (Figure 1E). There was a noticeable difference in the melting temperature (*T*_m_) curves of oroxylin A‐treated compared to control cells (Figure 1F). The average *T*_m_ increased from 64.8°C in control group to 71.0°C in oroxylin A‐treated group, indicating that oroxylin A triggered thermal stabilisation of TKT by direct binding. The results of DARTS and CETSA, used to validate drug target engagement, were highly consistent. The *K_D_* value was 11.9 μM for the interaction of oroxylin A with TKT determined by surface plasmon resonance (SPR) (Figure 1G).

To gain mechanistic insight into its binding to human TKT (PDB code 3MOS), oroxylin A was docked into the active site delineated by thiamine pyrophosphate (TPP). As shown in Figure 1H, the two hydroxyl groups (as donors) formed a crucial hydrogen bond with Gln189. The methoxy group (as an acceptor) formed a third hydrogen bond with Gln428. The bicyclic core was caged by two edge‐to‐face π‐interactions with His77 and His258. The phenyl ring extended into the binding channel of the TPP phosphate motif, blocking its chelation of calcium ion, an essential natural cofactor in TPP‐dependent enzymes. The binding affinity was predicted to be −6.5 kcal∙mol^‐1^, reflecting a modest binding potency.

Next, competitive displacement assay was conducted using TKT protein column and an available TKT inhibitor, oxythiamine. Results showed that the retention time of oxythiamine was markedly decreased with the increased concentration of oroxylin A in mobile phase (Figure 1I), indicating oroxylin A competed with the same receptor of oxythiamine. Moreover, aspartate 155 of TKT was reported to be essential for the enzyme activity. Then, the histidine 77 and aspartate 155 were mutated to evaluate the protection effects of oroxylin A on TKT from proteolysis. Results found oroxylin A obviously protected the wild‐type TKT protein from digestion, while TKT‐A77H and TKT‐A155D mutated proteins were almost fully digested into small peptides and amino acid (Figure 1J), revealing that histidine 77 and aspartate 155 were crucial binding sites of oroxylin A with TKT.

### 3.2 Oroxylin A inhibits hepatocellular carcinoma growth in vitro and in vivo

**FIGURE 2:**
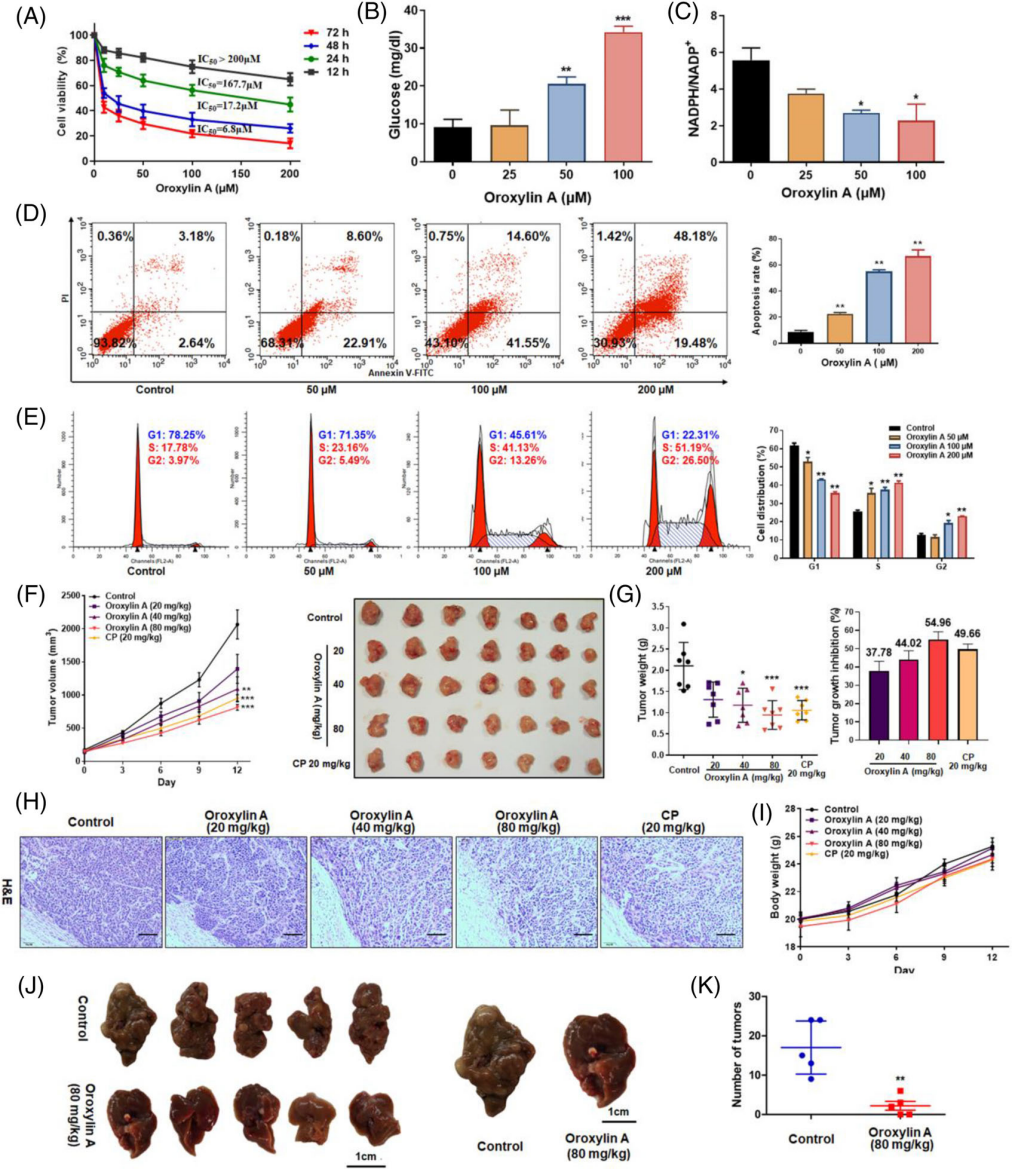
Oroxylin A inhibits hepatocellular carcinoma growth in vitro and in vivo. (A) HepG2 cell viability measured by Cell Counting Kit‐8 assay after exposure to 0‐200 μM oroxylin A for 12, 24, 48, and 72 h. Effects of oroxylin A on (B) glucose consumption and (C) NADPH/NADP^+^ ratio in HepG2 cells. (D) Apoptosis determined by annexin V‐FITC/PI staining and flow cytometry after exposure to various concentrations of oroxylin A for 48 h. (E) Cell cycle distribution determined by flow cytometry after exposure to 0‐200 μM oroxylin A for 24 h. Data are means ± SD; *p < 0.05 and **p < 0.01 versus negative control (*n* = 3). (F) Effect of oroxylin A on growth of HepG2 xenograft tumours. Tumour volume was measured using calipers every 3 days for 12 days, and photographs of tumours removed from animals. (G) Tumours were weighed (left panel) immediately after euthanasia and the growth inhibition rate was calculated (right panel). Data are presented as means ± SD; *p < 0.05, **p < 0.01, and ***p < 0.001 versus the negative control (*n* = 7). (H) H&E staining of xenograft tumours. Scale bar: 200 μm. (I) Body weight. (J) Macroscopic features of the liver of diethylnitrosamine‐treated mice in control and oroxylin A‐treated groups. (K) Number of liver tumours per mouse by microscopy.

TKT deficiency reduces cell proliferation, increases apoptosis, and alleviates hepatic steatosis and fibrosis.^15^ To examine the effect of oroxylin A on cell proliferation in vitro, SMMC‐7721, Huh‐7, HepG2, Hep3B, SK‐hep‐1, Hep1‐6, and L02 cells were treated with various concentrations of oroxylin A for 48 h and their proliferation was examined by Cell Counting Kit‐8 assay. Treatment with 25 μM oroxylin A decreased the viability of all of the cell lines by 35‐80%, with the greatest decrease seen in HepG2 cells and no obvious toxicity in L02 (Figure S1). In HepG2 cells, oroxylin A exerted an anti‐proliferative effect within 12 h, the magnitude of which increased for 72 h thereafter. Oroxylin A inhibited HepG2 cell proliferation in a time‐ and dose‐dependent manner, with IC_50_ values of 17.2 and 6.8 μM at 48 and 72 h, respectively (Figure 2A).

We found oroxylin A upregulated glucose in cell lyse, indicating reduced glucose consumption of cells (Figure 2B). Moreover, the NADPH/NADP^+^ ratio was dose‐dependently decreased with oroxylin A, representing reduced power in tumour cells (Figure 2C). To examine whether oroxylin A represses proliferation by inducing apoptosis or cell cycle arrest, HepG2 cells were treated with oroxylin A for 48 h, harvested, and analysed by flow cytometry. Indeed, the proportions of cells in early and late apoptosis were significantly increased from 5.8% to 31.5%, 56.1%, and 67.5%, respectively, by oroxylin A (Figure 2D). Additionally, cell cycle was arrested in S/G2 phase, and the proportion of cells in G1 phase was reduced in a dose‐dependent manner by oroxylin A (Figure 2E). Therefore, oroxylin A‐induced cell cycle arrest in S/G2 phase before cell death in HepG2 cells, suggesting that oroxylin A suppressed cell proliferation by inhibiting cell cycle progression. To verify our in vitro observation and assess the antitumour activity of oroxylin A in vivo, a human HepG2 tumour xenograft nude mouse model was established. Results indicated oroxylin A caused a significant decrease in tumour volume compared to vehicle (Figure 2F). Tumour weights were lower in oroxylin A‐treated than control mice (Figure 2G, left panel), and the average tumour growth inhibition rates of the 20, 40, and 80 mg·kg^‐1^·day^‐1^ treatment groups were 37.78%, 44.02%, and 54.96%, respectively, compared to the control group (Figure 2G, right panel). Histological analysis of tumour sections revealed that oroxylin A triggered much less hypercellular region and nuclear polymorphism of tumour cells, implying that oroxylin A induced obvious tumour apoptosis and necrosis in vivo (Figure 2H). Notably, the mouse body weights, reflecting general health, were not significantly different among the groups, indicating no acute toxicity at any concentration tested (Figure 2I). Moreover, in DEN‐induced HCC mice, small white nodules in the liver were macroscopically observed (Figure 2J). The number and size of white nodules in 80 mg·kg^‐1^ oroxylin A‐treated mice were significantly reduced. The tumour incidence was 60% in oroxylin A‐treated group compared to 100% in the control group (Table S1). The average number of tumours per liver was 2.2 in oroxylin A‐treated group compared to 17 in the control group, representing 87.06% reduction (Figure 2K). Therefore, oroxylin A demonstrated remarkable antitumour activity in vivo. Additionally, oroxylin A had no significant influence on glucose tolerance and insulin tolerance in mice (Figure S2A,B).

### 3.3 Oroxylin A suppresses transketolase and activates p53 signalling

**FIGURE 3:**
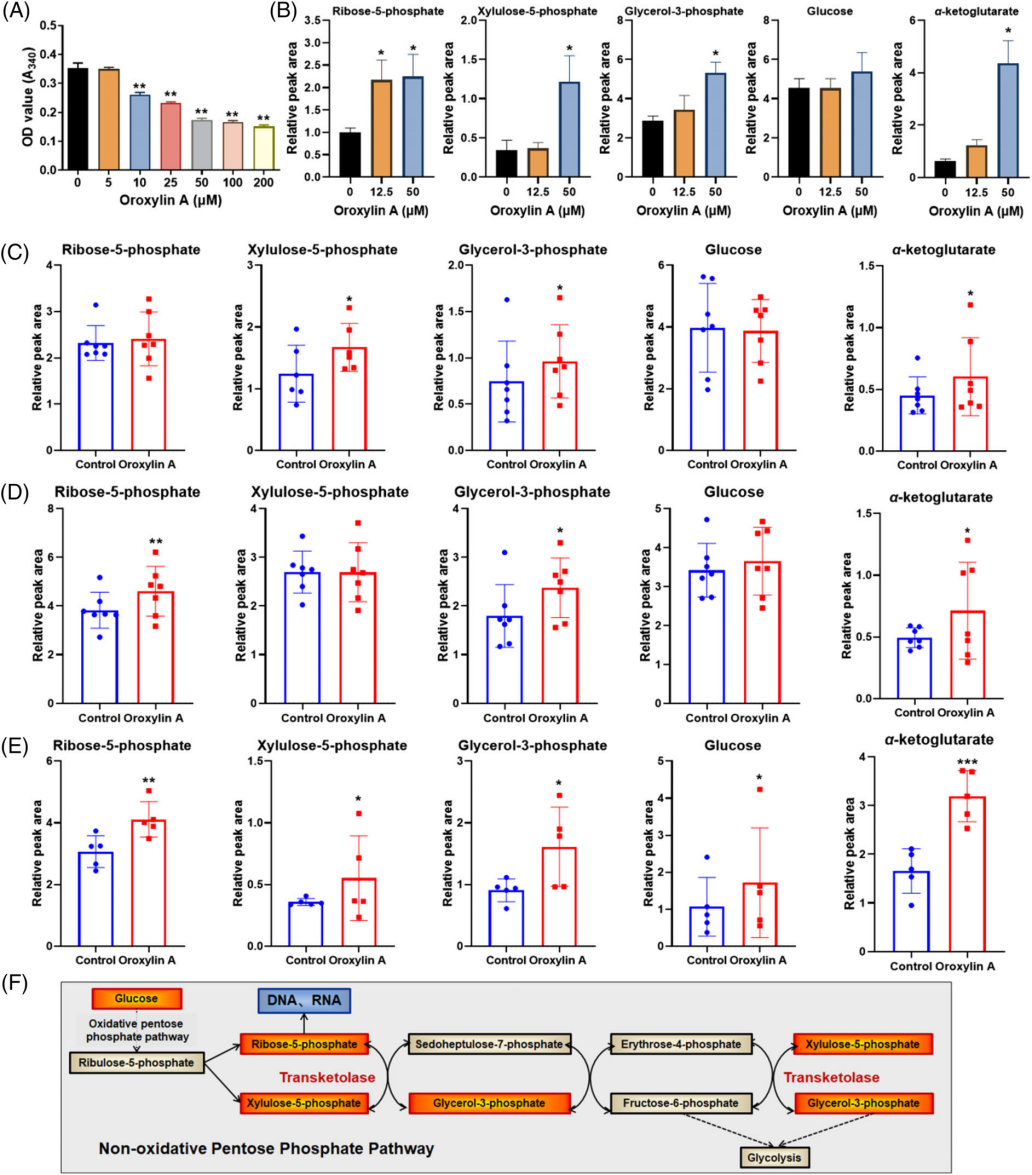
Oroxylin A suppresses transketolase (TKT) activity in vitro and in vivo. (A) In vitro TKT activity determined based on NAD^+^ reduction rate in the presence of oroxylin A (*n* = 3). (B) Levels of ribose‐5‐phosphate, xylulose‐5‐phosphate, glycerol‐3‐phosphate, glucose, and α‐ketoglutarate in control and oroxylin A‐treated HepG2 cells. Metabolite levels in the control and oroxylin A‐treated (C) HepG2 xenograft tumours, (D) SMMC‐7721 xenograft tumours, and (E) diethylnitrosamine (DEN)‐induced liver tumours. Data are means ± SD; *p < 0.05, **p < 0.01, and ***p < 0.001 *vs*. the negative control (*n* = 5). (F) Non‐oxidative PPP was inhibited by oroxylin A. Red represents accumulation, and blue represents abnormal control.

The interaction between TKT and oroxylin A might alter the activity of TKT. Therefore, TKT activity was examined in vitro in the presence of oroxylin A. Results indicated TKT activity was dose‐dependently reduced by oroxylin A (50% reduction by 50 μM oroxylin A; Figure 3A). TKT is a ubiquitous enzyme catalysing the reversible transfer of a dihydroxyethyl fragment from a ketose donor to an aldose acceptor. To evaluate the effect of TKT on cellular metabolism, metabolomic analysis by UPLC‐QTOF‐MS was performed in HCC cells, xenografts, and DEN‐induced tumours. Because glycolytic intermediates are typically of medium to high polarity, identification was achieved using a Waters Xbrigde Amide column and authentic standards (Figure S3). Interestingly, several intermediates including R5P, Xu5P, G3P, glucose, and α‐ketoglutarate were markedly accumulated in oroxylin A‐treated cells and tumours (Figure 3B–E), suggesting activity inhibition of TKT by oroxylin A. Therefore, activity suppression of TKT by oroxylin A led to accumulation of glycolytic intermediates in the non‐oxidative PPP (Figure 3F).

**FIGURE 4:**
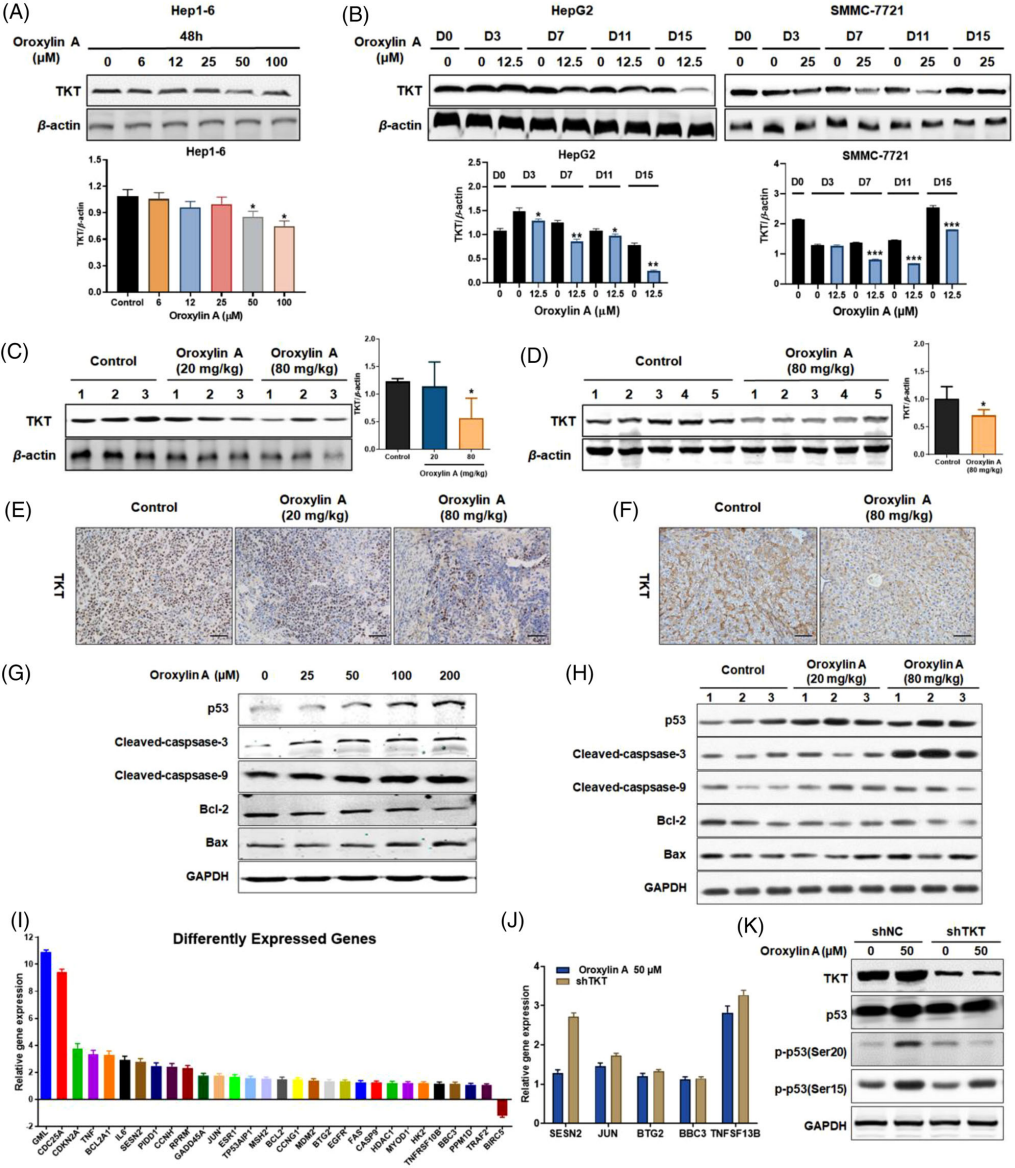
Oroxylin A significantly affects transketolase (TKT) protein expression and p53 signalling. (A) Western blotting analysis of TKT in Hep1‐6 cells treated with 0‐100 μM oroxylin A for 48 h. (B) Western blotting analysis of TKT in HepG2 and SMMC‐7721 cells treated with oroxylin A for 0‐15 days. Western blotting and immunohistochemistry of TKT in (C,E) HepG2 xenograft tumours, and in (D,F) diethylnitrosamine (DEN)‐treated mice. Scale bar: 200 μm. Western blotting of proteins involved in p53 signalling in (G) HepG2 cells and (H) HepG2 xenograft tumours. (I) Differently expressed mRNAs involved in p53 signalling of TKT‐knockdown HepG2 cells using RT‐PCR array (*n* = 3) and evaluated by (J) RT‐PCR analysis. (K) p53 expression and phosphorylation levels in HepG2 cells transfected with shNC or shTKT lentivirus treated with 0 or 50 μM oroxylin A for 48 h. Data are means ± SD; *p < 0.05 *vs*. the negative control (*n* = 3).

Next, western blotting showed oroxylin A dose‐dependently decreased TKT protein levels after 48 h treatment in Hep1‐6 mouse hepatoma cells (Figure 4A), whereas 14 days continuous treatment led to less TKT expression in HepG2 and SMMC‐7721 cells (Figure 4B). Correspondingly, TKT depletion was detected in HepG2 xenograft tumours and DEN‐induced liver tumours, which was confirmed by immunohistochemical staining (Figure 4C–F).

To map the comprehensive pathway changes regulated by oroxylin A, we described sequencing‐based transcriptome profiling, providing full‐length analysis of mRNA expression in HepG2 cells treated with 0 or 25 μM oroxylin A for 24 h. Totally, 578 genes were differentially expressed at least two fold after oroxylin A treatment, with 29 genes upregulated and 449 genes downregulated (Table S2; Figure S4A,B). Kyoto Encyclopedia of Genes and Genomes (KEGG) pathway enrichment analysis showed that hypoxia‐inducible factor‐1 (HIF‐1) signalling, glycosphingolipid biosynthesis, and several amino acid metabolism pathways closely correlated with the PPP were significantly influenced by oroxylin A. Notably, the most frequent pathway affected was p53 signalling (Figure S4C). As expected, oroxylin A treatment upregulated p53 level and key proteins of p53 signalling including cleaved‐caspase‐3, cleaved‐caspase‐9, and Bax while downregulated Bcl‐2 in HepG2 cells, HepG2 xenograft tumours, and DEN‐induced liver tumours (Figure 4G,H; Figure S4D). RT‐PCR array analysis of 84 genes involved in p53 signalling was performed on TKT‐knockdown HepG2 cells (Figure 4I). Thirty RNAs were differentially expressed, with 29 genes upregulated and 1 downregulated (fold changes > 2.0 or < 0.5, *p* < 0.05; Table S3). Several tumour suppressor genes were upregulated, including GML (glycosyl‐phosphatidylinositol anchored molecule‐like protein, 10.8‐fold), CDKN2A (cyclin‐dependent kinase inhibitor 2A, 3.8‐fold), TNF (tumour necrosis factor, 3.3‐fold), TP53AIP1 (TP53‐regulated apoptosis‐inducing protein 1, 1.6‐fold), and CASP9 (protein structure prediction round 9, 1.2‐fold), whereas the anti‐apoptotic gene BIRC5 (baculoviral IAP repeat containing 5) was downregulated (1.2‐fold). The results were evaluated by RT‐PCR (Figure 4J). Furthermore, TKT depletion reduced the oroxylin A‐mediated upregulation and phosphorylation of p53 (Figure 4K; Figure S4E). However, the binding potency of p53‐oroxylin A was weaker than that of TKT‐oroxylin A with a *K_D_* value of 70.7 μM determined by SPR (Figure S4F). These results demonstrated that oroxylin A not only inhibited TKT activity and expression, but also activated p53 signalling partially through targeting TKT.

### 3.4 TKT knockdown attenuates oroxylin A‐mediated HCC suppression

**FIGURE 5:**
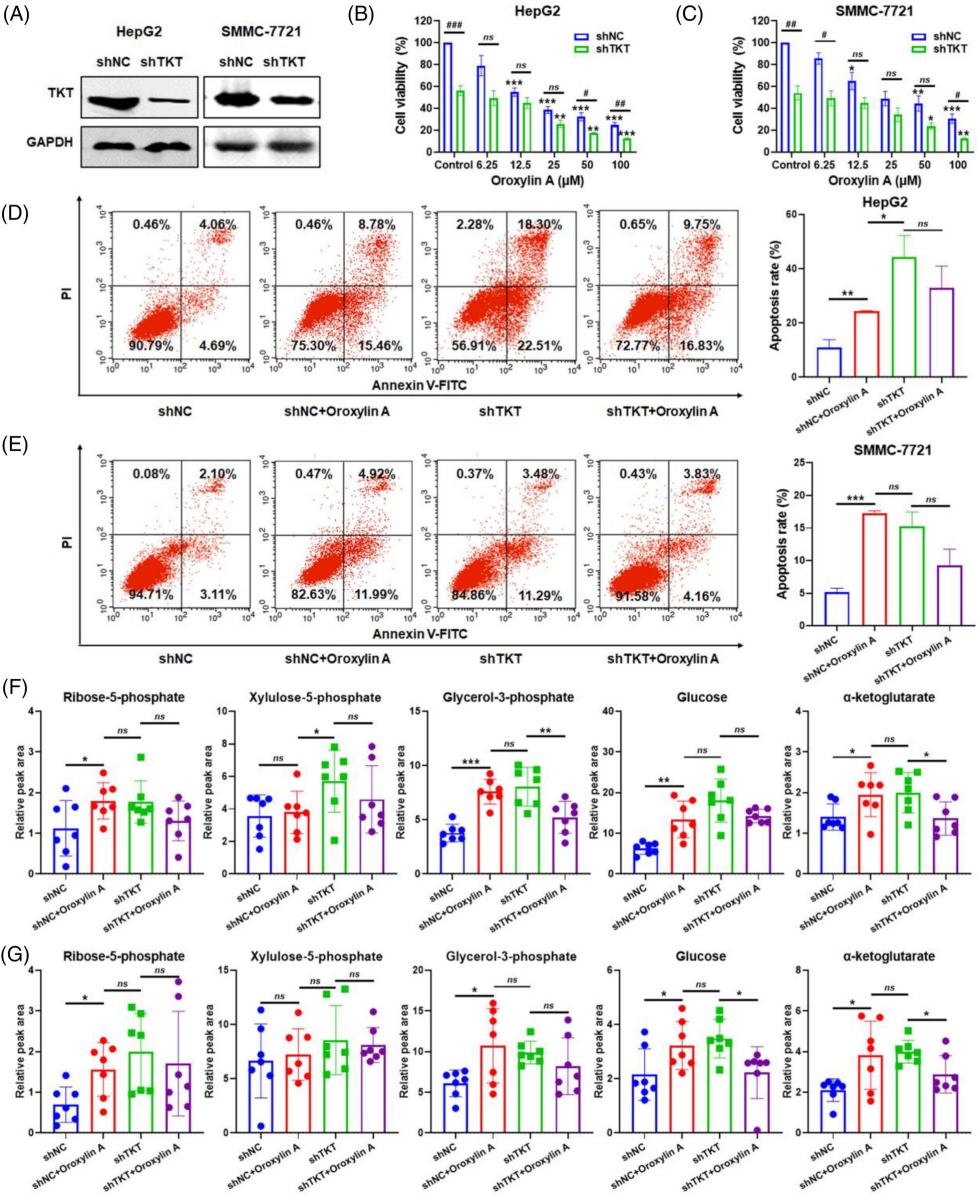
Transketolase (TKT) depletion increases resistance to oroxylin A in vitro. (A) Western blotting of the TKT protein level in HepG2 and SMMC‐7721 cells transfected with shNC or shTKT lentivirus after puromycin selection. Viability of (B) HepG2 cells and (C) SMMC‐7721 cells transfected with shNC or shTKT lentivirus after exposure to 0‐100 μM oroxylin A for 48 h. Data are means ± SD. ^#^ *p* < 0.05, ^##^ *p* < 0.01, ^###^ *p* < 0.001 *vs*. the shNC groups; *p < 0.05, **p < 0.01, ***p < 0.001 *vs*. non‐oroxylin A‐treated groups (*n* = 3). Apoptosis of (D) HepG2 cells and (E) SMMC‐7721 cells transfected with shNC or shTKT lentivirus, as determined by annexin V‐FITC/PI staining and flow cytometry with oroxylin A treated for 48 h. Levels of ribose‐5‐phosphate (R5P), xylulose‐5‐phosphate (Xu5P), glycerol‐3‐phosphate (G3P), glucose, and α‐ketoglutarate in (F) HepG2 cells and (G) SMMC‐7721 cells transfected with shNC or shTKT lentivirus with oroxylin A treated for 48 h. Data are means ± SD; *p < 0.05, **p < 0.01, ***p < 0.001 (*n* = 7).

To determine whether oroxylin A suppresses tumour growth by targeting TKT, stable TKT‐knockdown HepG2 and SMMC‐7721 cell lines were established (Figure 5A). TKT knockdown significantly decreased cell proliferation in vitro and attenuated oroxylin A‐induced cell growth inhibition. Cell viability was reduced from 38.48% to 25.74%, and 48.94% to 34.08% with 25 μM oroxylin A in HepG2 and SMMC‐7721 cells, respectively (Figure 5B,C). TKT depletion also promoted apoptosis, which was modulated slightly by oroxylin A (Figure 5D,E). By contrast, knockdown of TKT blocked oroxylin A‐mediated decrease of TKT activity, accompanied by decreased accumulation of R5P, Xu5P, G3P, glucose, and α‐ketoglutarate in HepG2 and SMMC‐7721 cells (Figure 5F,G).

**FIGURE 6:**
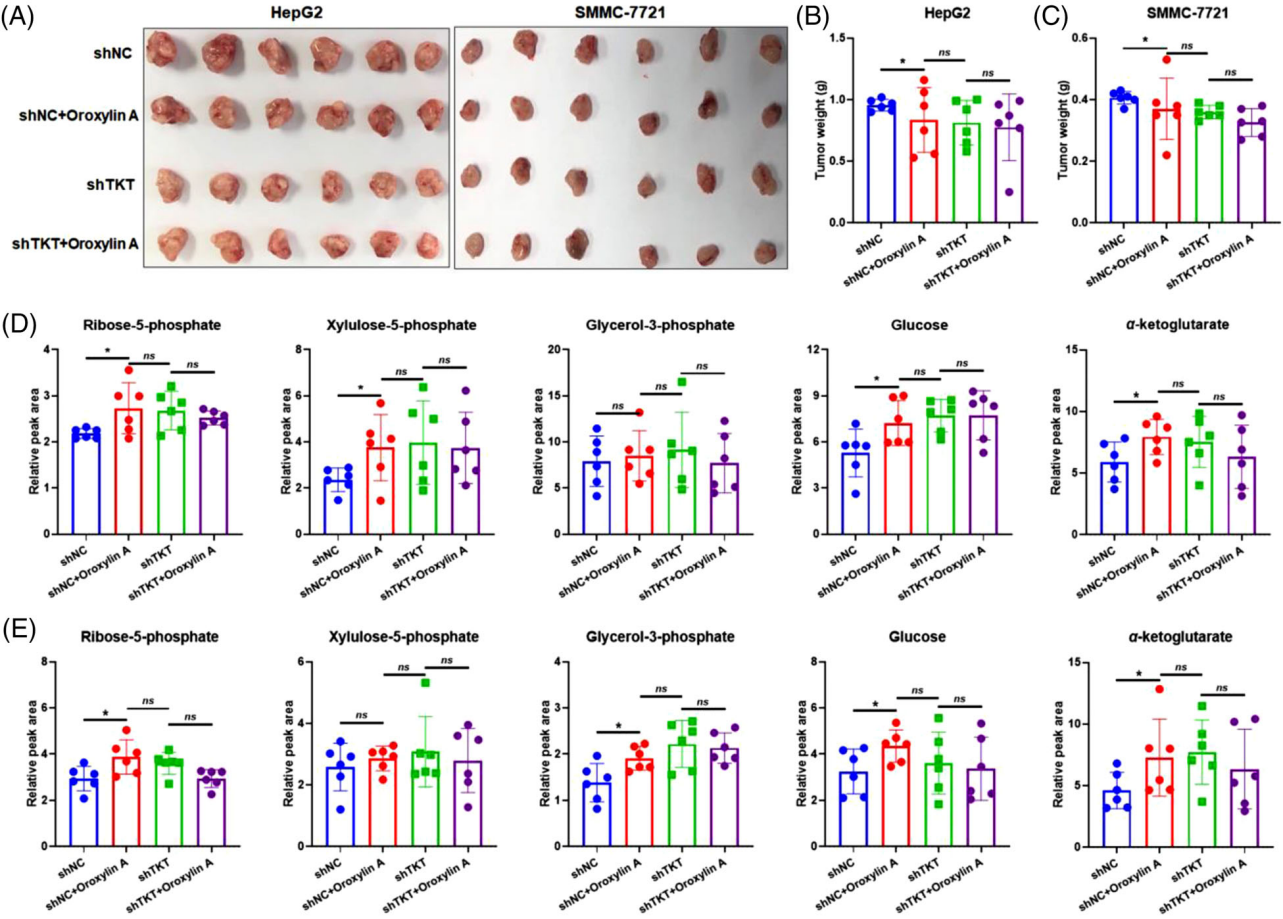
Transketolase (TKT) depletion increases resistance to oroxylin A in vivo. (A) Photographs of tumours removed from HepG2 and SMMC‐7721 xenograft animals transfected with shNC or shTKT lentivirus and treated with 80 mg·kg^−1^ oroxylin A. Tumour weight of (B) HepG2 and (C) SMMC‐7721 xenograft animals. Levels of ribose‐5‐phosphate (R5P), xylulose‐5‐phosphate (Xu5P), glycerol‐3‐phosphate (G3P), glucose, and α‐ketoglutarate in (D) HepG2 and (E) SMMC‐7721 xenograft tumours transfected with shNC or shTKT lentivirus. Data are means ± SD; *p < 0.05, **p < 0.01, and ***p < 0.001 *vs*. the control groups (*n* = 6).

To further evaluate the effect of TKT depletion on oroxylin A‐mediated HCC repression in vivo, stable TKT knockdown and negative control HepG2 and SMMC‐7721 cell lines were injected subcutaneously into nude mice (6 mice/group) to produce implanted tumours. Tumours in both TKT‐knockdown groups were significantly smaller than that of control groups (Figure 6A). Oroxylin A caused much less growth inhibition with TKT deletion (Figure 6B,C). Moreover, the accumulation of non‐oxidative PPP intermediates including R5P, Xu5P, G3P, and glucose as well as α‐ketoglutarate was reduced (Figure 6D,E). These data together suggested that TKT depletion impaired oroxylin A‐mediated HCC inhibition, further supporting TKT as a functional target of oroxylin A in HCC suppression.

### 3.5 Oroxylin A inhibits the growth of patient‐derived tumour organoids

**FIGURE 7:**
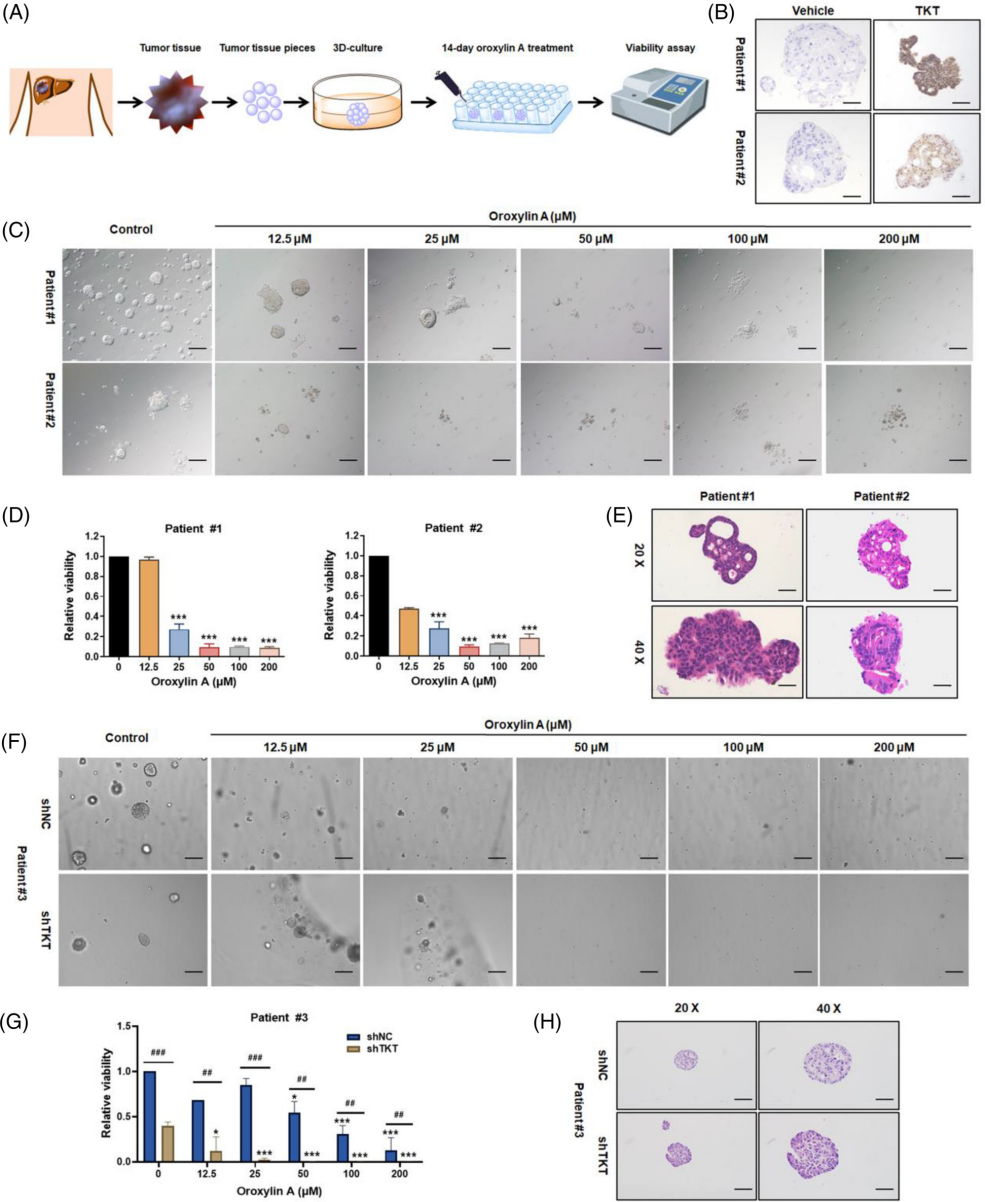
Oroxylin A inhibits the growth of patient‐derived organoids (PDOs). (A) Experimental design. (B) Immunohistochemistry of transketolase (TKT) in patient tissues. Scale bar: 100 μm. (C) Representative images of the growth of organoids treated with 0‐100 μM oroxylin A for 14 days. Scale bar: 100 μm. (D) Viability and (E) haematoxylin and eosin (H&E) staining of tissue sections of Patient #1 and Patient #2‐derived organoids treated with 0‐100 μM oroxylin A for 14 days. (F) Representative images, (G) viability, and (H) H&E staining of tissue sections of Patient #3‐derived organoids and shTKT lentivirus transfected organoids treated with 0‐100 μM oroxylin A for 14 days. Scale bar: 100 μm. Data are presented as means ± SD. ^#^ *p* < 0.05, ^##^ *p* < 0.01, ^###^ *p* < 0.001 *vs*. the shNC groups; *p < 0.05, **p < 0.01, ***p < 0.001 vs. non‐oroxylin A‐treated groups (*n* = 3).

We also generated PDOs and determined the effect of oroxylin A on their growth. The organoids were incubated for 2 weeks with 0, 12.5, 25, 50, 100, or 200 μM oroxylin A (Figure 7A). TKT expression in PDOs was confirmed by immunohistochemical staining (Figure 7B). The PDOs had a distinct microscopic appearance and culture behaviour during the treatment period, and the rate of cell death increased with increasing oroxylin A concentration. Also, the organoid reconstitution rate was significantly decreased by oroxylin A (Figure 7C). Oroxylin A suppressed organoid growth in a dose‐dependent manner, with the viability of organoids reduced by >50% at 50 μM (Figure 7D). These results were confirmed by the morphological similarities between PDOs and the biopsies from which they originated (Figure 7E). Coincidently, TKT depletion remarkably suppressed organoid growth which attenuated the oroxylin A‐mediated HCC inhibition effects (Figure 7F–H). Therefore, oroxylin A has therapeutic potential for cancer.

## 4 DISCUSSION

Reprogrammed metabolism is a hallmark of cancer. Several metabolic enzymes induce metabolic reprogramming, including glucose‐6‐phosphate‐dehydrogenase (G6PD),^23^ hypoxia‐inducible factor‐1α (HIF‐1α),^24^ and hexokinase 2 (HK2).^25^ TKT, a key enzyme of the non‐oxidative PPP, plays a crucial role in carbohydrate transformation by supplying cancer cells with R5P for ribonucleotide synthesis. TKT can determine the direction of metabolite flux in the non‐oxidative PPP in a temporal manner due to its reversible nature. TKT depletion was reported to promote HIF‐1α degradation via α‐ketoglutarate signalling, attenuate NADPH provision, increase the levels of reactive oxygen species (ROS), and decrease glucose flux and the levels of purine metabolites.^4^, ^26^ TKT promoted HCC development in both non‐metabolic and metabolic respects via its nuclear localisation.^15^ Therefore, TKT may be a crucial target for metabolic reprogramming of tumours. However, due to the poor efficacy or unpredictable side effects, only a few TKT inhibitors (oxythiamine, thiamine) currently are available for basic research.^27^

Herbal medicines are sources of new, safe, and effective drugs for cancer.^28^, ^29^ Oroxylin A had diverse pharmacological functions and marked potency, and was a potent anticancer agent in drug‐containing rat serum after oral administration of *R. scutellariae* with low toxicity to normal tissue.^30^, ^31^ Pharmacokinetic study revealed oroxylin A conjugated rapidly and extensively with glucuronic acid, and eliminated rapidly in rat plasma after administration.^32^ Of note, the table of oroxylin A has been approved for phase Ⅰ clinical trial for treatment of HCC by National Medical Products Administration (NMPA; ChiCTR2100051434). These suggested oroxylin A might be a promising clinical‐applied agent for drug resistance reversal.

Numerous reports proved that the anticancer effect of oroxylin A was mediated by the induction of apoptosis, inhibition of invasion and metastasis, overcoming of multi‐drug resistance, and suppression of angiogenesis by several cancer cell lines.^33^ Oroxylin A could also effectively suppress glycolysis.^34^, ^35^ Our findings suggested that oroxylin A was a potent TKT inhibitor, and also inhibited non‐oxidative PPP and HCC tumour growth in mice and PDOs for the first time. Intriguingly, the promising inhibitor also exhibited excellent therapeutic efficacy against TKT‐overexpressed tumours (Figure S5).

Transcriptome profiling was carried out to gain greater insights into the underlying antitumour mechanisms of oroxylin A and investigate whether the PPP or related pathways was influenced. KEGG enrichment analysis revealed several pathways closely correlated with the PPP were affected by oroxylin A. HIF‐1 was regarded as the primary factor mediating glucose metabolism and cell survival under low oxygen conditions as in tumour environments.^36^ Glycosphingolipid synthesis was reported to be highly dependent on NADPH and uridine diphosphate (UDP) glucose, two products of the PPP.^37^, ^38^ Correspondingly, previous reports suggested that oroxylin A activated, upregulated, and stabilised p53 in various tumour cells, thus inhibiting the PPP and affecting nucleotide synthesis.^39^, ^40^, ^41^, ^42^ Additionally, multiple siRNAs were used in gene‐silencing experiments to reduce the off‐target effects in this study.

Given above, we hypothesised that TKT inhibition may lead to p53 signalling activation resulted from abnormal nucleic acids synthesis as R5P accumulation. TKT depletion activated p53 signalling and reduced the oroxylin A‐induced upregulation and phosphorylation of p53. This confirmed that oroxylin A directly targeted TKT and inhibited the non‐oxidative PPP, possibly further activated p53 signalling. Studies revealed that TKT knockdown caused R5P accumulation by affecting the provision of NADPH to counteract ROS, as well as promoting genome instability during liver injury and tumour initiation.^4^, ^16^ Additionally, p53 was found to directly bind G6PD, the first rate‐limiting enzyme of the oxidative PPP, and inhibit its activity.^43^ This leads to suppression of the diversion of glycolytic intermediates into the PPP, possibly enhancing the anticancer effect of oroxylin A. However, TKT and p53 were not physically associated by co‐IP. Taken together, our results uncover an unexpected role of oroxylin A in TKT inhibition for the first time, which may offer new opportunities for the discovery of new scaffolds and new application of TKT inhibitors as well as cancer therapy.

## CONFLICT OF INTEREST

The authors declare no conflict of interest.

## Supporting information

Supporting Information.docx

Supporting Information Table S2.xlsx
