# Supplementary material for: Novel transketolase inhibitor oroxylin A suppresses the non‐oxidative pentose phosphate pathway and hepatocellular carcinoma tumour growth in mice and patient‐derived organoids
Source: Clin Transl Med. 2022 Oct 31;12(11):e1095. doi: 10.1002/ctm2.1095 (PMC9619225; doi:10.1002/ctm2.1095)
Supplement: Supplementary file 1 — Supporting Information.docx [file CTM2-12-e1095-s001.docx]

**Supporting Information**

**Novel transketolase inhibitor oroxylin A suppresses the non-oxidative pentose**

**phosphate pathway and hepatocellular carcinoma tumor growth in mice and patient-derived organoids**

Dan Jia^1,2,3,4,#^, Chunliang Liu^1,#^, Zhenyu Zhu^2,#^, Yan Cao^2,#^, Wen Wen^1,3^, Zhanying Hong^2^, Yue Liu^2^, Erdong Liu^1,3^, Long Chen^2^, Chun Chen^2,5^, Yanqiu Gu^2,5^, Binghua Jiao^4^, Yifeng Chai^2^, Hong-yang Wang^1,3,*^, Jing Fu^1,3,*^, Xiaofei Chen^1,^^2,3,*^

^1^International Cooperation Laboratory on Signal Transduction, Eastern Hepatobiliary Surgery Hospital, Second Military Medical University/Naval Medical University, Shanghai, China

^2^School of Pharmacy, Second Military Medical University/Naval Medical University, Shanghai, China

^3^National Center for Liver Cancer, Second Military Medical University/Naval Medical University, Shanghai, China

^4^Department of Biochemistry and Molecular Biology, College of Basic Medical, Second Military Medical University/Naval Medical University, Shanghai, China

^5^Department of Pharmacy, Shanghai Ninth People’s Hospital, School of Medicine of Shanghai Jiao Tong University, Shanghai, China

^#^Equal contributors.

^*^Corresponding authors.

HongyangWang and Jing Fu, International Cooperation Laboratory on Signal Transduction, Eastern Hepatobiliary Surgery Hospital, Naval Medical University, 225 Changhai Road, Shanghai 200438, China.

Xiaofei Chen, School of Pharmacy, Second Military Medical University/Naval Medical University, 325 Guohe Road, Shanghai, China.

E-mail addresses: fujing-724@163.com (J. Fu), hywangk@sina.vip.com (H.-Y. Wang), [xfchen2010@163.com](mailto:xfchen2010@163.com) (X. Chen)

**Supplementary Methods**

**Cell proliferation/apoptosis/cycle assay**

Cell proliferation assay was carried out according to the protocol for cell counting kit**-**8 (CCK-8) assay supplied by the manufacturer (Dojindo Laboratories, Kumamoto, Japan). After 24 h incubation, cells were treated with various concentrations of oroxylin A for 12, 24, 48 and 72 h. Then, 10 μL of CCK**-**8 solution was added to each well and incubated at 37°C for 1.5 h. The absorbance was read at 450 nm on a microplate absorbance reader (Bio**-**RAD instruments, USA).

Apoptosis was quantified by flow cytometry using an Annexin VFITC apoptosis detection kit I (BD PharmingenTM CA, USA). 5 × 10^5^ cells were plated and treated with various concentrations of oroxylin A for 48 h. Then, cells were harvested, washed twice in PBS, and stained with Annexin V**-**FITC and propidium iodide (PI) for 10min at room temperature in the dark. Finally, the cells were analyzed with a flow cytometer (Becton Dickinson) and WinMDI 2.8 software (Scripps Institute, La Jolla, CA, USA).

Cell cycle distribution was analyzed with a FACScalibur flow cytometer (BD Biosciences). 5 × 10^5^ HepG2 cells were plated and treated with various concentrations of oroxylin A for 48 h. Then, cells were harvested, washed twice in PBS, resuspended in 70% (v/v) cold ethanol and stored at −20°C overnight. Next, cells were harvested, washed twice in PBS and incubated with RNase A at 37°C for 30 min followed by stained with PI at 4°C solution in the dark for 30min. Cell cycle distribution was analyzed by BD CellQuest Pro software (BD Biosciences).

**Targeted quantitative metabolomics analysis**

The metabolomics platform employed in the present study has been previously described in detail. Briefly, when HepG2 cells in 100-mm plates reached approximately 70% confluent, the supernatant was replaced by fresh medium with 0, 12.5 and 50 µM oroxylin A. After 24 h incubation, the culture medium was removed and cells were washed three times with cold Milli-Q water and quenched with liquid nitrogen. For extraction, 1.8 mL of ice cold MeOH/CHCl3/Milli-Q water (4:1:1) containing 5 μg/μL L-2-chlorophenylalanine as internal standard was added to each plate. Cells were scraped with a cell lifter, transferred to 2 mL micro-centrifuge tubes, ultrasonicated in an ice bath ultrasonicator for 15 min and subsequently centrifuged at 16,000 g for 15 min at 4°C. Supernatants were collected and lyophilized using a Heto Power Dry LL3000 freeze drier (Thermo Electron Co., Bath, UK). The residues were resuspended in 100 μL of MeOH/ACN/Milli-Q water mixture (1:1:1), vortexed and centrifuged at 16,000 g for 15 min at 4°C. The supernatants were transferred into glass auto-samplers and stored at −80°C prior to analysis. In parallel a quality control (QC) sample was prepared by mixing equal volumes of 10 μL from each samples, and injected five times at the beginning of the run to ensure system equilibration and then every five samples to further monitor the stability of the analysis.

An XBridge^TM^ Amide column (3.0 × 100 mm, 3.5 μm, Waters, Milford, MA) was used for separation. The mobile phase consisted of H_2_O modified with 0.1% formic acid (A) and ACN modified with 0.1% formic acid (B), using a gradient elution of 95% B at 0-2 min, 95-65% B at 2-10 min, 65-50% B at 10-13 min and 50% B at 13-15 min. The total run time was 20 min including equilibration. The flow rate was 400 μL/min. An electrospray ionization source interface was used and set in positive mode. The following parameters were employed: capillary voltage, 3.5 kV; drying gas flow, 11 L/min; gas temperature, 350°C; nebulizer pressure, 45 psig; fragmentor voltage, 120 V; skimmer voltage, 60 V. Data were collected in centroid mode and the mass range was set at *m/z* 50–1000 using extended dynamic range. The MS/MS spectra of metabolites were obtained by a collision energy ramp from 10 to 30 eV.

**Glucose Tolerance Test**

Mice were intraperitoneally administered with saline or 80 mg/kg oroxylin A every 2 days for 2 weeks. After the last administration, the mice were fasted for 14 h. 10% Glucose (1 g/kg) was administered by intraperitoneal (i.p.) injection, and blood glucose levels were measured at 0, 15, 30, 60, 90 and 120 min using a glucometer.

**Insulin Tolerance Test**

Mice were intraperitoneally administered with saline or 80 mg/kg oroxylin A every 2 days for 2 weeks. After the last administration, the mice were fasted for 4 h. 0.2U/mL insulin (0.75U/kg) was administered by intraperitoneal (i.p.) injection, and blood glucose levels were measured at 0, 15, 30, 60, 90 and 120 min using a glucometer.

**mRNA sequencing by Illumina HiSeq**

After 24h incubation, HepG2 cells were approximately 80% conﬂuent and treated with 50 µM oroxylin A or the vehicle control (0.5% DMSO) for 12 h. Total RNA of each sample was quantified and qualified by Agilent 2100 Bioanalyzer (Agilent Technologies, Palo Alto, CA, USA), NanoDrop (Thermo Fisher Scientific Inc.) and 1% agrose gel. 1 μg total RNA with RIN value above 7 was used for following library preparation. Next generation sequencing library preparations were constructed according to the manufacturer’s protocol (NEBNext® Ultra™ RNA Library Prep Kit for Illumina®). The poly (A) mRNA isolation was performed using NEBNext Poly(A) mRNA Magnetic Isolation Module (NEB). The mRNA fragmentation and priming was performed using NEBNext First Strand Synthesis Reaction Buffer and NEBNext Random Primers.

First strand cDNA was synthesized using ProtoScript II Reverse Transcriptase and the second-strand cDNA was synthesized using Second Strand Synthesis Enzyme Mix. The purified double-stranded cDNA (by AxyPrep Mag PCR Clean-up (Axygen)) was then treated with End Prep Enzyme Mix to repair both ends and add a dA-tailing in one reaction, followed by a T-A ligation to add adaptors to both ends. Size selection of Adaptor-ligated DNA was then performed using AxyPrep Mag PCR Clean-up (Axygen), and fragments of ~360 bp (with the approximate insert size of 300 bp) were recovered. Each sample was then amplified by PCR for 11 cycles using P5 and P7 primers, with both primers carrying sequences which can anneal with flow cell to perform bridge PCR and P7 primer carrying a six-base index allowing for multiplexing. The PCR products were cleaned up using AxyPrep Mag PCR Clean-up (Axygen), validated using an Agilent 2100 Bioanalyzer (Agilent Technologies, Palo Alto, CA, USA), and quantified by Qubit 2.0 Fluorometer (Invitrogen, Carlsbad, CA, USA).

Then libraries with different indices were multiplexed and loaded on an Illumina HiSeq instrument according to manufacturer’s instructions (Illumina, San Diego, CA, USA). Sequencing was carried out using a 2 × 150 bp paired-end (PE) configuration; image analysis and base calling were conducted by the HiSeq Control Software (HCS) + OLB + GAPipeline-1.6 (Illumina) on the HiSeq instrument.

**Quality Control**

In order to remove technical sequences, including adapters, polymerase chain reaction (PCR) primers, or fragments thereof, and quality of bases lower than 20, pass filter data of fastq format were processed by Trimmomatic (v0.30) to be high quality clean data.

**Mapping**

Firstly, reference genome sequences and gene model annotation files of relative species were downloaded from genome website, such as UCSC, NCBI, ENSEMBL. After index reference genome sequence, clean data were aligned to reference genome via software Hisat (v2.0.14).

**Expression analysis**

A count table for differential expression analysis was prepared by software HTSeq (v0.6.1). As a stand-alone script, htseq-count counts for each gene how many aligned reads overlap its exons, and its input file contains a SAM/BAM file and a GTF or GFF file with gene models.

**Differential expression analysis**

Differential expression analysis used the DESeq Bioconductor package, a model based on the negative binomial distribution. After adjusted by Benjamini and Hochberg’s approach for controlling the false discovery rate, P-value of genes were setted < 0.05 to detect differential expressed ones.

**GO and KEGG enrichment analysis**

GO-TermFinder was used identifying Gene Ontology (GO) terms that annotate a list of enriched genes with a significant pvalue less than 0.05. KEGG (Kyoto Encyclopedia of Genes and Genomes) is a collection of databases dealing with genomes, biological pathways, diseases, drugs, and chemical substances (http://en.wikipedia.org/wiki/KEGG). We used scripts in house to enrich significant differential expression gene in KEGG pathways.

**Novel transcripts prediction**

After assembling a transcriptome via software Stringtie (V1.0.4) from one or more samples, Cuffcompare, a tool of Cufflinks v2.2.1, compared assembly to known transcripts. Then, novel transcripts can be predicted via results of Cuffcompare.

**Alternative splicing analysis**

Asprofile v1.0 is a suite of programs for extracting, quantifying and comparing alternative splicing (AS) events from RNA-seq data. It took a GTF transcript file created by Cufflinks as its input.

**SNV analysis**

Samtools v0.1.18 with command mpileup and Bcftools v0.1.19 were used to do SNV calling.

**Supplementary figures**


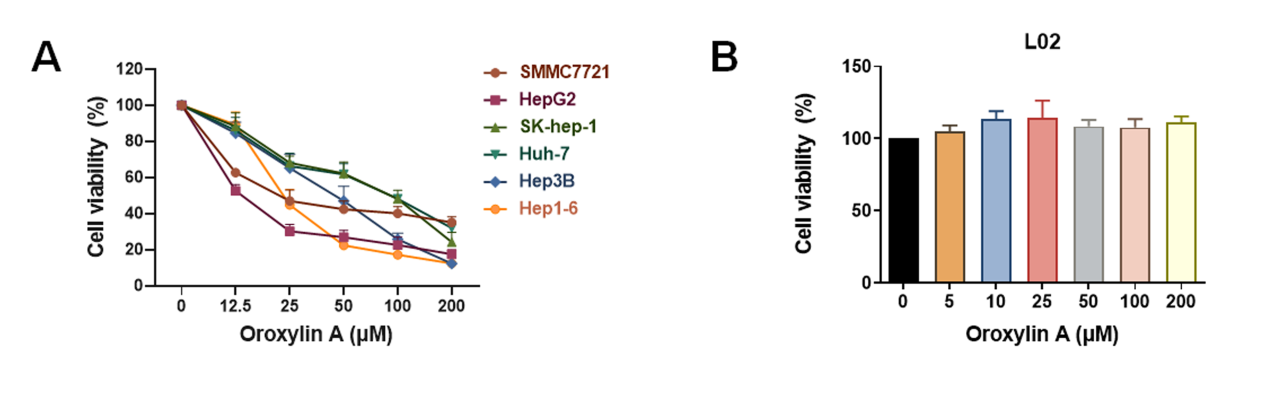


**Figure S1** (A) Cell viability of SMMC-7721, Huh-7, HepG2, Hep3B, SK-hep-1, and Hep1-6 measured by Cell Counting Kit-8 assay after exposure to 0-200 μM oroxylin A for 12, 24, 48, and 72 h. (B) Cell viability of L02 cells after exposure to 0-200 μM oroxylin A for 48 h.

.


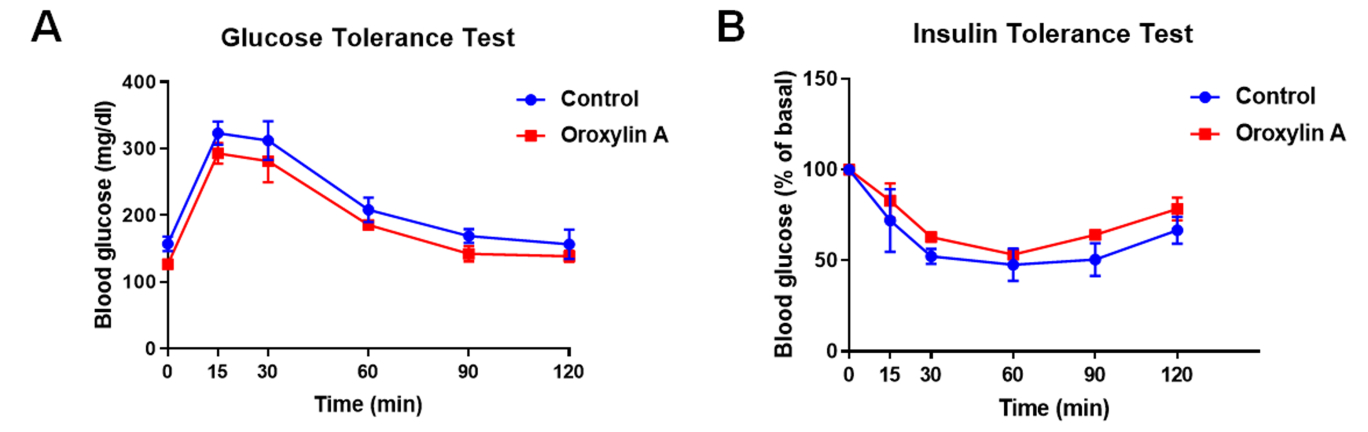


**Figure S2** Effects of oroxylin A on (A) glucose tolerance, and (B) insulin tolerance in mice. Data are presented as means ± SD (n = 5)


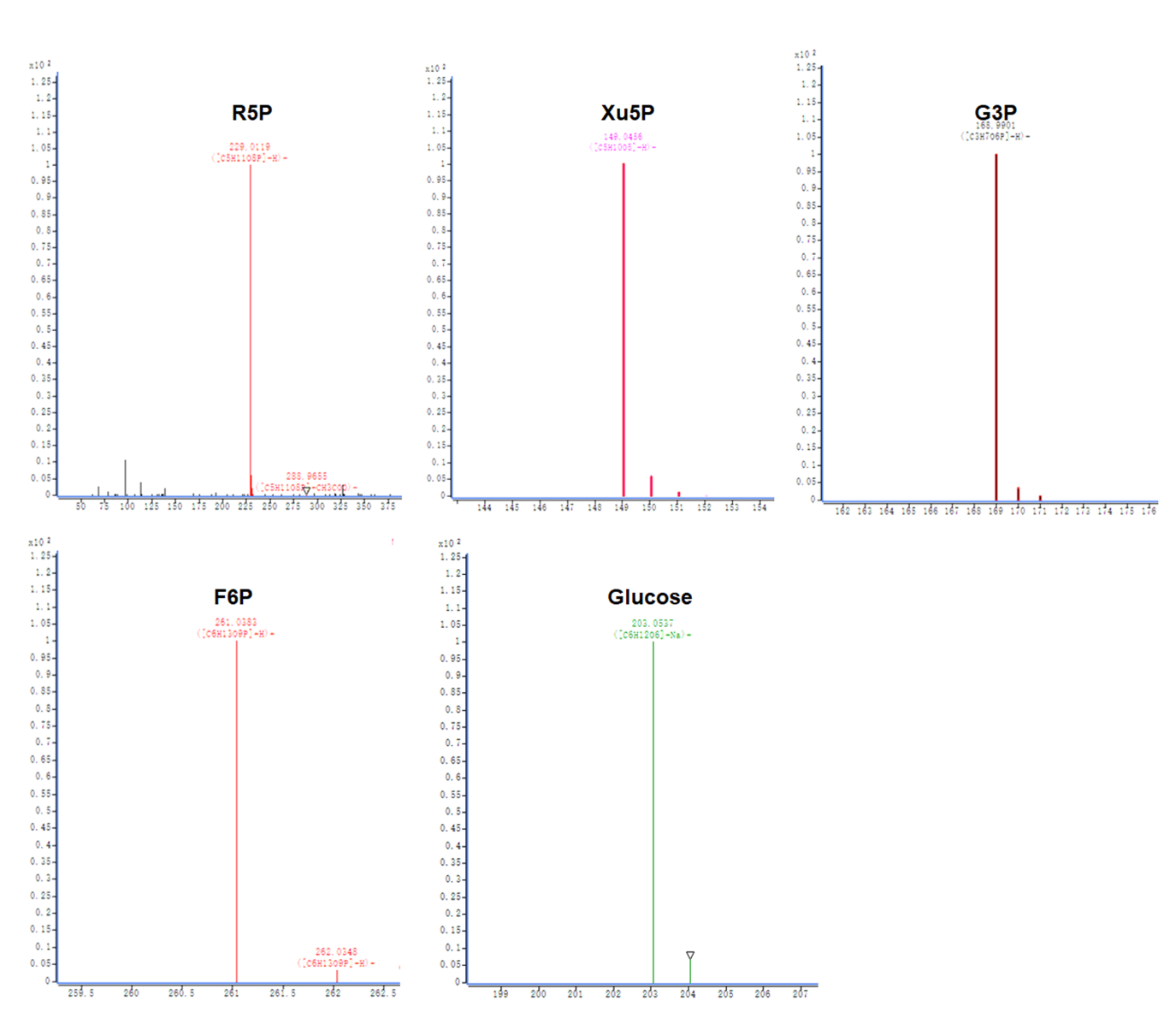


**Figure S3** Typical mass spectrum of ribose-5-phosphate (R5P), xylulose-5-phosphate (Xu5P), glycerol-3-phosphate (G3P), fructose-6-phosphate (F6P), and glucose.


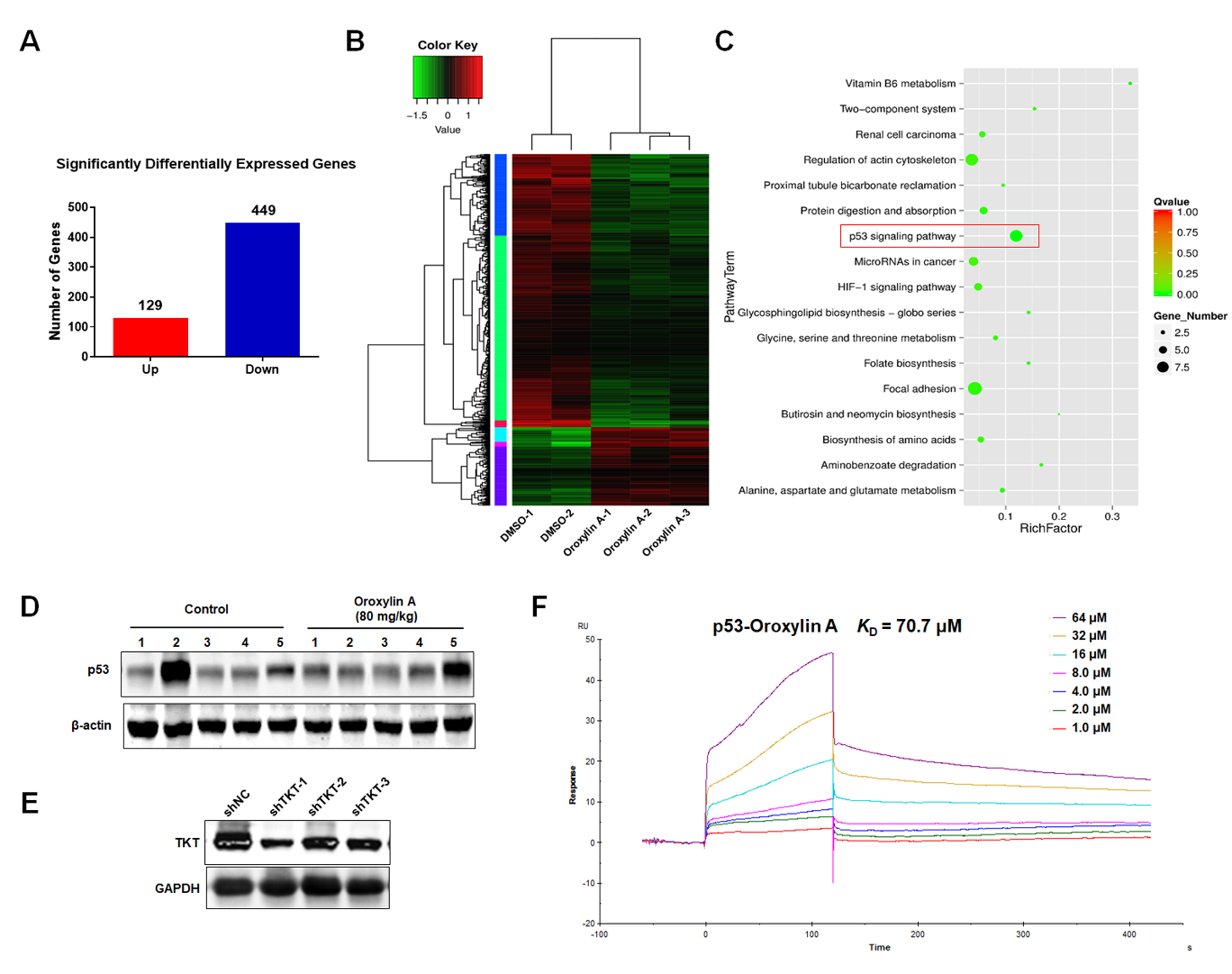
**Figure S4** Transcriptome analysis of HepG2 cells treated with 50 µM oroxylin A or vehicle control (0.5% DMSO) for 12 h. (A) The number of significant differentially expressed genes (DEGs). DEseq2 algorithms were used to detect the DEGs. Y axis represents DEG numbers. Red color represents upregulated DEGs and blue color represents downregulated DEGs. (B) Heat map representation of unsupervised hierarchical clustering showed gene expression ratios of each sample. X axis represents the sample. Y axis represents the different expression genes. The color represents the log2RPKM transformed gene expression level. Red color means high expression level while green color means low expression level. (C) Pathway functional enrichment of different expression genes. X axis represents enrichment factor. Y axis represents pathway name. The color indicates the Q-value (high: red, low: green), the lower Q-value indicates the more significant enrichment. Point size indicates different expression gene number (bigger dots refer to larger amount). Rich Factor refers to the value of enrichment factor, which is the quotient of foreground value (number of different expression genes) and background value (total gene amount). The larger the value, the more significant enrichment. (D) Western blotting of p53 protein level in DEN-treated rat liver with or without oroxylin A treatment. (E) Protein levels of TKT in different HCC stable cell lines were evaluated by western blotting. (F) Surface plasmon resonance analysis of oroxylin A and p53 immobilized on a chip.


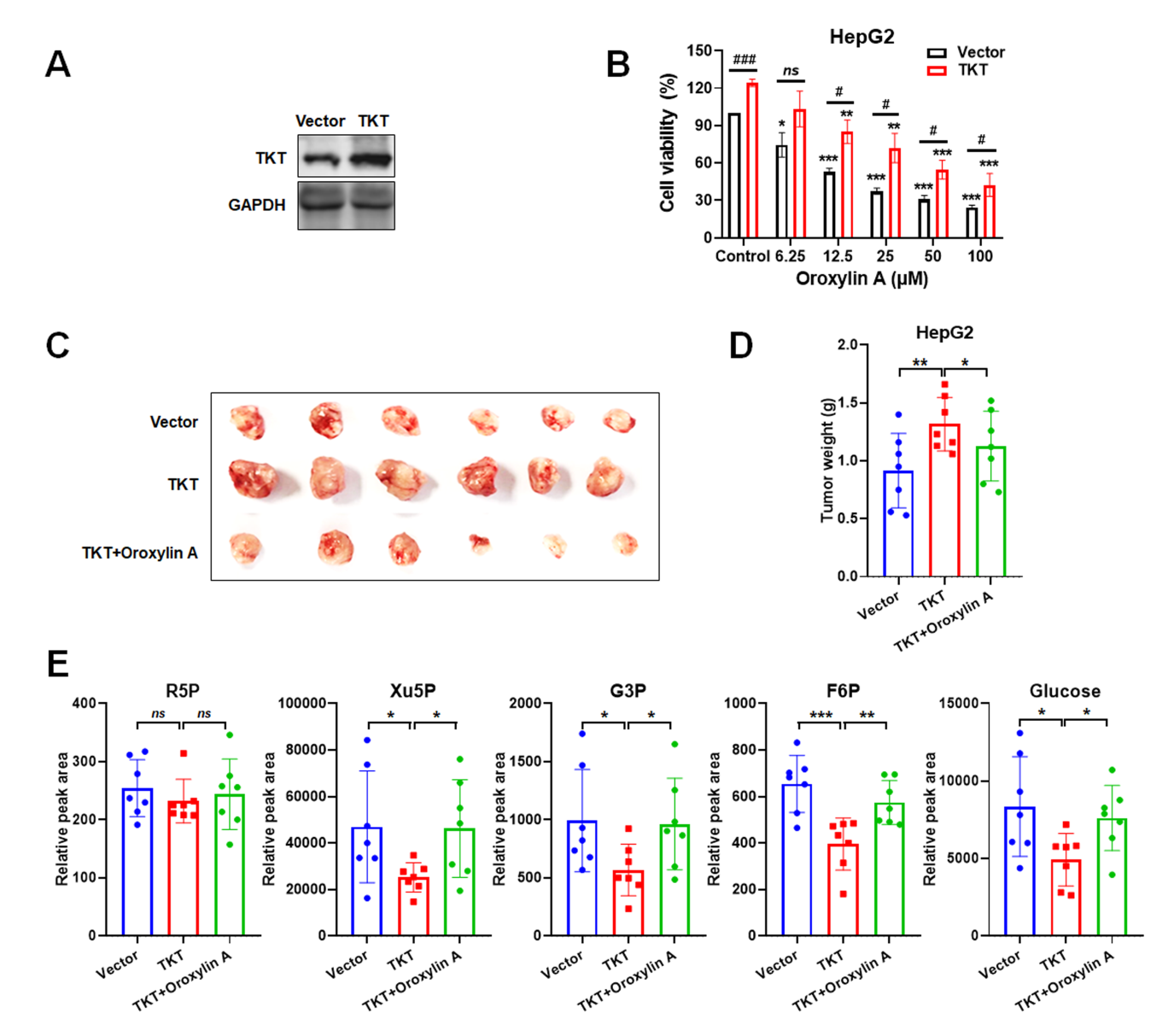


**Figure S5** Anti-tumor effects of oroxylin A on TKT-overexpressed tumor. (A) Western blotting of theTKT protein level in HepG2 cell transfected with Vector or TKT lentivirus after puromycin selection. (B) Viability of HepG2 cell cells transfected with vector or TKT lentivirus after exposure to 0-100 μM oroxylin A for 48 h. Data are means ± SD; *P < 0.05, **P < 0.01, ***P < 0.001 *vs.* non-oroxylin A treated groups; ^#^P < 0.05, ^##^P < 0.01, ^###^P < 0.001 vs. the vector groups; (n = 3). (C) Photographs of tumors removed from HepG2 xenograft animals transfected with Vector or TKT lentivirus and treated with 80 mg/kg oroxylin A. (D) Tumor weight of HepG2 xenograft animals transfected with Vector or TKT lentivirus and treated with 80 mg/kg oroxylin A. (E) Levels of ribose-5-phosphate (R5P), xylulose-5-phosphate (Xu5P), glycerol-3-phosphate (G3P), fructose-6-phosphate (F6P), and glucose in HepG2 xenograft tumors transfected with Vector or TKT lentivirus, and treated with 80 mg/kg/d oroxylin A. Data are means±SEM; *P < 0.05, **P < 0.01, and ***P < 0.001 *vs.* the negative control (n = 7).

**Supplementary tables**

**Table S1.** Summary of the number of liver tumors per mouse DEN-treated rats.

| Control | 24 | 24 | 15 | 13 | 9 |
| --- | --- | --- | --- | --- | --- |
| **Oroxylin A**  **(80 mg/kg)** | 6 | 3 | 2 | 0 | 0 |

**Table S3** Analysis of the expression of RNAs in p53 signaling (n = 3).

| **No.** | **Gene** | **siRNA(-)** | **siRNA(+)** | **Log2FDR** |
| --- | --- | --- | --- | --- |
| 1 | GML | 1.12089E-05 | 0.039623496 | 11.7875 |
| 2 | CDC25A | 1.32928E-05 | 0.009449249 | 9.473415362 |
| 3 | CDKN2A | 8.92369E-05 | 0.001204546 | 3.754705371 |
| 4 | TNF | 6.56852E-06 | 6.4833E-05 | 3.303088823 |
| 5 | BCL2A1 | 2.12516E-06 | 2.03588E-05 | 3.260008841 |
| 6 | IL6 | 6.09929E-05 | 0.000457148 | 2.905949541 |
| 7 | SESN2 | 2.17282E-06 | 1.45434E-05 | 2.742728318 |
| 8 | PIDD1 | 1.82721E-05 | 0.000100343 | 2.45722043 |
| 9 | CCNH | 0.002231145 | 0.011885779 | 2.413380281 |
| 10 | RPRM | 3.45951E-06 | 1.68559E-05 | 2.284610689 |
| 11 | GADD45A | 0.000161296 | 0.000543042 | 1.751350229 |
| 12 | JUN | 0.003239529 | 0.010815657 | 1.739265137 |
| 13 | ESR1 | 3.10925E-06 | 9.85457E-06 | 1.66422464 |
| 14 | TP53AIP1 | 4.83853E-06 | 1.42177E-05 | 1.555050772 |
| 15 | MSH2 | 0.020025749 | 0.056688316 | 1.501195231 |
| 16 | BCL2 | 0.000421553 | 0.001186129 | 1.492476156 |
| 17 | CCNG1 | 0.003393524 | 0.009325898 | 1.4584583 |
| 18 | MDM2 | 0.001555938 | 0.004094469 | 1.395892029 |
| 19 | BTG2 | 0.002224968 | 0.00561471 | 1.335427214 |
| 20 | EGFR | 0.028596849 | 0.07094903 | 1.31092677 |
| 21 | FAS | 0.001058326 | 0.002541246 | 1.263751755 |
| 22 | CASP9 | 0.002431395 | 0.005725222 | 1.235547253 |
| 23 | HDAC1 | 0.002211131 | 0.005118608 | 1.210967103 |
| 24 | MYOD1 | 1.32461E-06 | 3.02197E-06 | 1.189918967 |
| 25 | HK2 | 0.00039169 | 0.000889545 | 1.183354963 |
| 26 | TNFRSF13B | 0.005648173 | 0.012659744 | 1.164392029 |
| 27 | BBC3 | 7.11848E-06 | 1.58712E-05 | 1.156770723 |
| 28 | PPM1D | 0.000454636 | 0.000952653 | 1.067237748 |
| 29 | TRAF2 | 0.000805404 | 0.001663401 | 1.046351602 |
| 30 | BRCA2 | 0.001687379 | 0.003306702 | 0.970608781 |
| 31 | BAX | 0.000495097 | 0.00095957 | 0.954675483 |
| 32 | MCL1 | 0.011156281 | 0.021161663 | 0.923596802 |
| 33 | TSC1 | 0.001194813 | 0.002241671 | 0.907790051 |
| 34 | PTEN | 0.006564951 | 0.012158304 | 0.88908579 |
| 35 | CRADD | 0.001132715 | 0.00201602 | 0.831725715 |
| 36 | SIAH1 | 0.000917503 | 0.001628146 | 0.827445436 |
| 37 | EI24 | 0.009131072 | 0.01613647 | 0.821468846 |
| 38 | TP53 | 0.002034655 | 0.003540308 | 0.799090744 |
| 39 | MYC | 0.144385723 | 0.246417216 | 0.771174958 |
| 40 | STAT1 | 0.030227385 | 0.051252191 | 0.761757503 |
| 41 | TP53BP2 | 0.002335586 | 0.00393623 | 0.753030341 |
| 42 | CDKN1A | 0.001872265 | 0.003033972 | 0.696423377 |
| 43 | ATR | 0.000854282 | 0.001382763 | 0.694769126 |
| 44 | CHEK1 | 0.008704638 | 0.013998493 | 0.685415362 |
| 45 | XRCC5 | 0.036803837 | 0.057995326 | 0.656080454 |
| 46 | APAF1 | 0.000521155 | 0.000817223 | 0.649016867 |
| 47 | FADD | 0.000258419 | 0.000402938 | 0.640847233 |
| 48 | KRAS | 0.003601965 | 0.005596041 | 0.635622288 |
| 49 | PCNA | 0.039968299 | 0.061757954 | 0.627768777 |
| 50 | MLH1 | 0.013276328 | 0.019583655 | 0.560793863 |
| 51 | NF1 | 0.014013877 | 0.020532551 | 0.551056718 |
| 52 | SIRT1 | 0.001595252 | 0.002328064 | 0.545346303 |
| 53 | E2F3 | 0.001492555 | 0.002151692 | 0.527687187 |
| 54 | NFKB1 | 0.001793508 | 0.002514706 | 0.487605659 |
| 55 | RB1 | 0.002424663 | 0.003324266 | 0.455251755 |
| 56 | TNFRSF10D | 0.000750427 | 0.001023829 | 0.448191045 |
| 57 | EGR1 | 0.007839623 | 0.010436644 | 0.412801738 |
| 58 | BID | 0.000939382 | 0.001156133 | 0.299523161 |
| 59 | MDM4 | 0.002972721 | 0.003649941 | 0.296088911 |
| 60 | CDK4 | 0.005806965 | 0.007121107 | 0.294317212 |
| 61 | WT1 | 0.000425958 | 0.000520807 | 0.290036446 |
| 62 | CHEK2 | 0.013387218 | 0.016225228 | 0.277382603 |
| 63 | CDK1 | 4.1947E-06 | 5.05055E-06 | 0.267870511 |
| 64 | PRC1 | 0.064883704 | 0.077992581 | 0.265480706 |
| 65 | PTTG1 | 0.012700244 | 0.015202218 | 0.259425626 |
| 66 | ATM | 0.012149157 | 0.014541313 | 0.259301402 |
| 67 | CCNB1 | 0.038687197 | 0.045579852 | 0.236540065 |
| 68 | RELA | 0.001242964 | 0.001452472 | 0.224725955 |
| 69 | DNMT1 | 0.004749528 | 0.005466714 | 0.202889516 |
| 70 | PRKCA | 0.00191028 | 0.002159642 | 0.177008018 |
| 71 | TADA3 | 0.003700664 | 0.004041488 | 0.127102348 |
| 72 | TP73 | 4.08E-06 | 4.34507E-06 | 0.090811398 |
| 73 | E2F1 | 0.000946572 | 0.000976223 | 0.04449899 |
| 74 | FASLG | 2.57143E-06 | 2.6273E-06 | 0.031014051 |
| 75 | KAT2B | 0.000696784 | 0.000703429 | 0.013692531 |
| 76 | IGF1R | 0.001996931 | 0.001980922 | -0.011612518 |
| 77 | BRCA1 | 0.00467441 | 0.004431908 | -0.076856382 |
| 78 | FOXO3 | 0.005150756 | 0.004582796 | -0.168556076 |
| 79 | CASP2 | 0.002068786 | 0.001678879 | -0.301285809 |
| 80 | TP63 | 3.89755E-06 | 3.15606E-06 | -0.304443088 |
| 81 | CCNE1 | 0.000169081 | 0.000133109 | -0.345106418 |
| 82 | CDC25C | 6.25773E-05 | 4.44305E-05 | -0.494090727 |
| 83 | BIRC5 | 0.000782295 | 0.000338651 | -1.20791421 |
